# Large-Scale microRNA Expression Profiling Identifies Putative Retinal miRNA-mRNA Signaling Pathways Underlying Form-Deprivation Myopia in Mice

**DOI:** 10.1371/journal.pone.0162541

**Published:** 2016-09-13

**Authors:** Andrei V. Tkatchenko, Xiaoyan Luo, Tatiana V. Tkatchenko, Candida Vaz, Vivek M. Tanavde, Sebastian Maurer-Stroh, Stefan Zauscher, Pedro Gonzalez, Terri L. Young

**Affiliations:** 1 Department of Ophthalmology, College of Physicians and Surgeons, Columbia University, New York, New York, United States of America; 2 Department of Pathology and Cell Biology, College of Physicians and Surgeons, Columbia University, New York, New York, United States of America; 3 Department of Ophthalmology, School of Medicine, Duke University, Durham, North Carolina, United States of America; 4 Center for Human Genetics, School of Medicine, Duke University, Durham, North Carolina, United States of America; 5 Bioinformatics Institute, Agency for Science Technology and Research, Singapore, Singapore; 6 Institute for Medical Biology, A*STAR, Singapore, Singapore; 7 Department of Mechanical Engineering and Materials Science, Pratt School of Engineering, Duke University, Durham, North Carolina, United States of America; 8 Department of Ophthalmology and Visual Sciences, School of Medicine and Public Health, University of Wisconsin, Madison, Wisconsin, United States of America; Ospedale Pediatrico Bambino Gesu, ITALY

## Abstract

Development of myopia is associated with large-scale changes in ocular tissue gene expression. Although differential expression of coding genes underlying development of myopia has been a subject of intense investigation, the role of non-coding genes such as microRNAs in the development of myopia is largely unknown. In this study, we explored myopia-associated miRNA expression profiles in the retina and sclera of C57Bl/6J mice with experimentally induced myopia using microarray technology. We found a total of 53 differentially expressed miRNAs in the retina and no differences in miRNA expression in the sclera of C57BL/6J mice after 10 days of visual form deprivation, which induced -6.93 ± 2.44 D (p < 0.000001, n = 12) of myopia. We also identified their putative mRNA targets among mRNAs found to be differentially expressed in myopic retina and potential signaling pathways involved in the development of form-deprivation myopia using miRNA-mRNA interaction network analysis. Analysis of myopia-associated signaling pathways revealed that myopic response to visual form deprivation in the retina is regulated by a small number of highly integrated signaling pathways. Our findings highlighted that changes in microRNA expression are involved in the regulation of refractive eye development and predicted how they may be involved in the development of myopia by regulating retinal gene expression.

## Introduction

Myopia is the most common vision disorder and a leading cause of visual impairment worldwide [1]. The prevalence of myopia has increased from 25% to 44% of the adult population in the United States in the last 30 years [2], and reached more than 80% of young adults in many parts of East Asia [3, 4]. In addition to its direct impact on visual acuity and quality of life, myopia is a major risk factor for potentially blinding ocular disorders such as cataract, glaucoma, retinal detachment, and myopic maculopathy, and represents one of the leading causes of blindness [5–7].

Postnatal refractive eye development is a tightly coordinated process, which is regulated by visual input [8]. Visual experience modulates ocular growth during early postnatal period and drives it towards sharp vision (i.e., perfect match between the eye’s optical power and its axial length) in a process called emmetropization [9]. During emmetropization, optical defocus evokes a signaling cascade that originates in the retina, propagates through other ocular tissues (i.e., retinal pigment epithelium and choroid), and results in scleral wall remodeling with increased eye growth. This signaling is associated with large-scale changes in gene expression in all ocular tissues, which was extensively studied at the mRNA level in several animal models of myopia [10–17]. Although these studies established that modulation of gene expression plays an important role in refractive eye development, non-coding transcriptome changes underlying refractive eye development have been largely unexplored.

MicroRNAs (miRNAs) are small non-coding RNAs that direct post-transcriptional regulation of gene expression by either facilitating degradation of their target mRNAs or suppressing mRNA translation [18–20]. MiRNAs often serve as nodes in signaling networks and modulate many cell activities, including cell proliferation, cell differentiation, metabolism, and synaptic function [21–24]. The potential influence of miRNAs on various biological processes is immense, as at least 50% of all coding genes are thought to be regulated by 1193 annotated miRNAs in mice and 1881 miRNAs in humans [22, 23, 25]. Insights into the full range of biologic functions of miRNAs are recent, and their involvement in disease has generated significant interest due to strong potential for therapeutic development [24, 26, 27].

MiRNA transcriptomes have been profiled in several ocular tissues [28] and miRNAs have been found to be expressed in the cornea, lens, retina, retinal pigment epithelium (RPE), and sclera of both embryonic and adult eyes [29–36]. A growing number of studies have shown that miRNAs play key roles in regulating both eye development and eye diseases [32, 34, 37–55]. For example, miR-96, miR-183, miR-1, and miR-133 have been implicated in retinitis pigmentosa [49], while miR-31, miR-150, and miR-184 have been associated with choroidal neovascularization [50] and diabetic retinopathy [51]. Mutations in miR-184 cause EDICT syndrome, familial keratoconus with cataract, and sporadic keratoconus [52, 53]. Mutations in the binding site of miR-328 within 3’-UTR of a myopia-causing gene *Pax6* were also shown to be associated with high myopia in a Chinese cohort [54, 55], suggesting that miRNAs play an important role in refractive eye development as well.

In the current study, we performed large-scale miRNA expression profiling in a mouse model of form-deprivation myopia and discovered extensive changes in miRNA expression in the myopic retina. The identified differentially expressed miRNAs were used to perform analysis of the miRNA-mRNA interaction networks in the myopic retina using a database of mRNAs differentially expressed in the myopic eye. This analysis revealed multiple mRNA targets for differentially expressed miRNAs and putative signaling pathways involved in the development of myopia.

## Materials and Methods

### Animals

C57BL/6J mice were obtained from the Jackson Laboratory (Bar Harbor, ME) and were maintained as an in-house breeding colony. C57BL/6J mice were recently shown not to have Rd8 mutation causing retinal degeneration in C57BL/6N mice [56]; however, C57BL/6J mice are known to have a relatively high incidence of microphthalmia, which affects from 4.4% to 10% of animals [57, 58]. Therefore, animals were screened for the presence of microphthalmia and other ophthalmic abnormalities such as corneal opacities and anterior polar cataracts often associated with this condition [59]. Each animal was first examined visually, which is often sufficient to identify animals with microphthalmia or cataract. If no anomalies are identified after visual examination, eyes were examined under the dissecting microscope and using handmade slit lamp. This was followed by the examination of the pupil image with the photorefractor. Animals found to have microphthalmia, corneal opacities or cataract were removed from the study (~10% in our colony). All procedures adhered to the ARVO Statement for the Use of Animals in Ophthalmic and Vision Research and were approved by the Columbia University Institutional Animal Care and Use Committee.

### Induction of Form-Deprivation Myopia

Visual form deprivation (VFD) was induced in 12 mice by applying a unilateral frosted hemispherical plastic diffusers over the right eyes as previously described [60]. Briefly, frosted hemispherical plastic diffusers were hand-made using caps from 0.2 ml PCR tubes (Molecular BioProducts, San Diego, CA) and rings made from medical tape (inner diameter 6 mm; outer diameter 8 mm). A cap was frosted with fine sandpaper and attached to a ring with Loctite™ Super Glue (Henkel Consumer Adhesives, Avon, OH). On the first day of the experiment (P24), animals were anesthetized via intraperitoneal injection of ketamine (90 mg/kg) and xylazine (10 mg/kg), and diffusers were attached to the skin surrounding the right eye with several stitches using size 5–0 ETHILON™ microsurgical sutures (Ethicon, Somerville, NJ) and reinforced with Vetbond™ glue (3M Animal Care Products, St. Paul, MN). The contralateral untreated left eyes were used as control. Toenails were covered with adhesive tape to prevent mice from removing the diffusers. Animals recovered on a warming pad and were then housed in transparent plastic cages for the duration of the experiment (10 days). A control group comprised of 8 age-matched C57BL/6J mice was maintained under the same experimental conditions as experimental group, but without VFD.

### RNA Extraction

After 10 days of visual form deprivation, mice were euthanized following the approved experimental protocol. Both myopic and control eyes were enucleated, cleaned by removing surrounding tissues and the crystalline lens, retina and sclera were dissected, snap-frozen in liquid nitrogen and stored in RNA*later*®-ICE (Life Technologies, Grand Island, NY). In order to obtain sufficient amount of tissue for RNA isolation, the retinas or scleras from three myopic or control eyes were pooled for RNA extraction. Three replicates (3 eyes per replicate) were processed in parallel. Total RNA was isolated from the retina and sclera samples using mirVana^TM^ miRNA isolation kit (Life Technologies, Grand Island, NY) according to the manufacturer’s instructions. RNA concentration was measured using NanoDrop 8000 spectrophotometer (Thermo Scientific, Wilmington, DE). The quality of the total RNA was assessed using Agilent RNA 6000 Nano kit and 2100 Agilent Bioanalyzer (Agilent Technologies, Santa Clara, CA) following the manufacturer’s instructions.

### MicroRNA expression profiling and microarray data analysis

MiRNA expression profiling was carried out at the Microarray Core Facility of the Duke University Institute for Genome Sciences and Policy (IGSP) using Agilent Mouse microRNA Microarray (release 15.0) (Agilent Technologies, Santa Clara, CA). MiRNA labeling, hybridization and washing were carried out according to the manufacturer’s recommendations. Following hybridization, microarrays were scanned on a DNA microarray scanner (Agilent G2565BA) and features were extracted using the Agilent Feature Extraction (AFE) image analysis tool (version A.9.5.3) with default protocols and settings. Gene expression data were analyzed using Partek Genomics Suit 6.6. Data were adjusted to bring the minimal signal to 0.5, normalized using quantile normalization procedure, and log2-transformed. This was followed by the removal of absent features and outliers. The normalized data were then analyzed using ANOVA to identify the differences in miRNA expression levels between myopic and control eyes. Differentially expressed miRNAs were identified using an FDR-adjusted *P*-value threshold of 0.05 and a cutoff of 2-fold change in expression. Differential expression was calculated as fold change (FC, myopic samples vs control).

### Analysis of miRNA-mRNA Signaling Pathways

To identify biologically relevant miRNA-regulated genetic networks, differentially expressed miRNAs were analyzed using QIAGEN’s Ingenuity Pathway Analysis (IPA^®^) software and database (QIAGEN, Redwood City, CA). Putative mRNA targets were identified for the differentially expressed miRNAs, and then the mRNAs were filtered using published datasets of mRNAs differentially expressed in the myopic retina [10, 13, 14, 61, 62]. Mouse orthologs of mRNAs found to be differentially expressed in the species other than mouse were identified using Ensembl Compara [63]. The list of miRNAs and their associated target mRNAs were then subjected to core functional analysis in IPA^®^ that uses ~1.5 million microRNA targeting interactions from sources including miRecords, TarBase, TargetScan, Ingenuity Expert findings and Ingenuity ExpertAssist findings to identify the miRNA-mRNA target relationships. Specifically, TargetScanMouse 6.2 [64], which was integrated with the latest versions of miRBase [65] and RefSeq [66, 67] databases, was used to identify mRNA targets. The stringency level for miRNA-mRNA interactions was set at “High (predicted)” or “Experimentally Observed”. “High (predicted)” confidence level was assigned to the relationships between a highly conserved microRNA and at least one conserved site on the targeted mRNA with the total TargetScan context score of -0.4 or less, which indicated that the microRNA was predicted to repress the expression of its mRNA target to at least 40% of the "normal" level. The “Experimentally Observed” targeting interactions were high quality manually curated miRNA-mRNA interactions with documented experimental support for each interaction. The homolog adjustment was applied to all miRNAs, which represent the miRNA families that share the same seed region, and IPA^®^ mouse miRNA cluster symbols were used in the network images for each miRNA. Significant and enriched networks and Gene Ontology categories were obtained using the Fisher's exact test with a *P*-value threshold of 0.05.

## Results

### MicroRNA expression profiling in the mouse form-deprivation myopia

**Fig 1:**
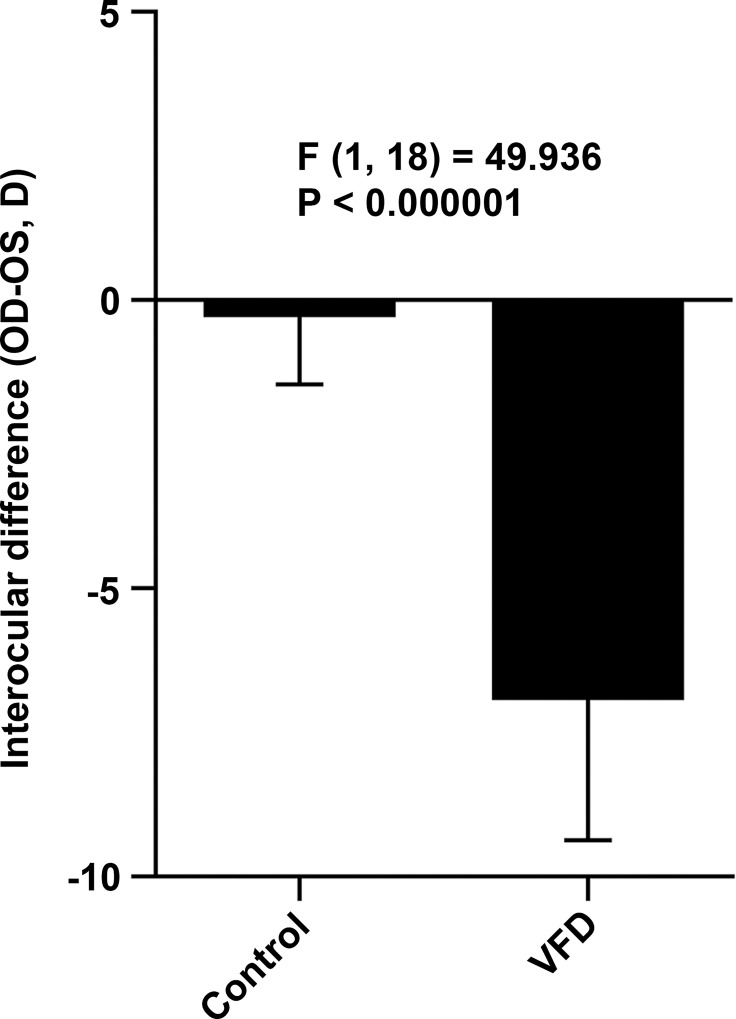
Form-deprivation myopia in C57BL/6J mice. Form-deprivation myopia was induced in C57BL/6J mice by applying a diffuser to the right eye of P24 animals. Ten days of visual form deprivation induced -6.93 ± 2.44 D (p < 0.000001, n = 12) of myopia in the right eyes compared to the contralateral control eyes.

**Fig 2:**
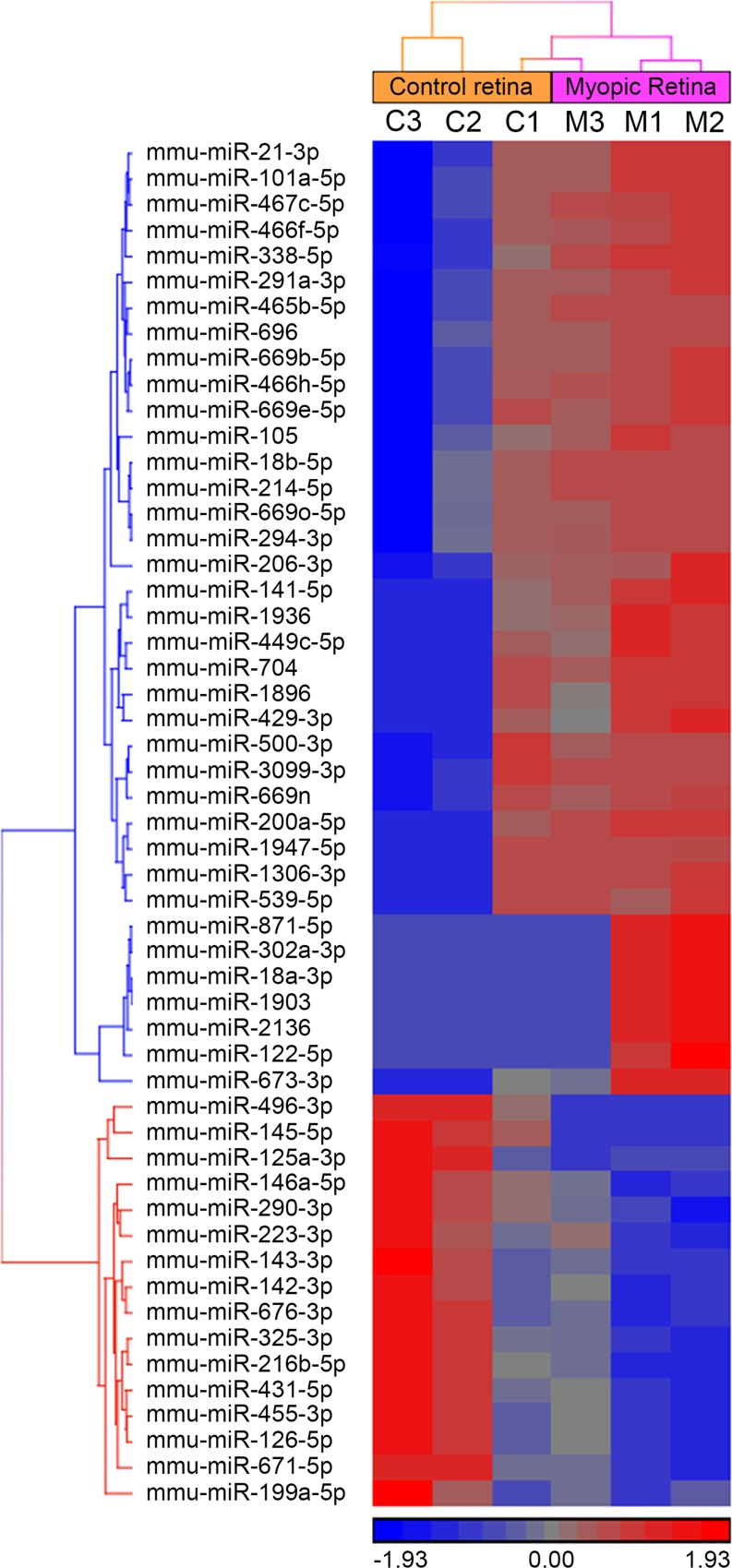
Hierarchical cluster analysis of 53 miRNAs differentially expressed in the myopic retina versus control retina. Logarithmic values (base 2) of Agilent total gene signal for differentially expressed miRNAs (cutoff: FC > 2, FDR-adjusted p-value < 0.05) were quantile normalized, shifted to mean of zero, scaled to standard deviation of 1.0 and subjected to hierarchical clustering using Euclidean dissimilarity and average linkage. The color scale indicates transcript abundance relative to the mean of zero: red identifies an increase in relative miRNA abundance; blue identifies a decrease in relative miRNA abundance. Columns show individual samples, whereas rows show individual miRNAs. Control samples c1, c2, and c3 correspond to myopic samples m1, m2, and m3 respectively. The “co-clustering” of the control sample c1 and myopic sample m3 resulted from the clustering algorithm that was used to generate the cluster and reflects individual differences in gene expression as well as differences in the myopic response to visual form deprivation between animals. It appears that the myopic response in the animals comprising experimental group 1 (samples c1 and m1) was the weakest among the 3 groups. However, the relationship between control sample c1 and the corresponding myopic sample m1 follows the same pattern as in the other two experimental groups even though samples c1 and m1 fall within the same color scheme. Consistent up- or down-regulation of the specific miRNAs in the myopic eyes versus corresponding control eyes across all samples is reflected by the low p-values shown in Table 1.

**Fig 3:**
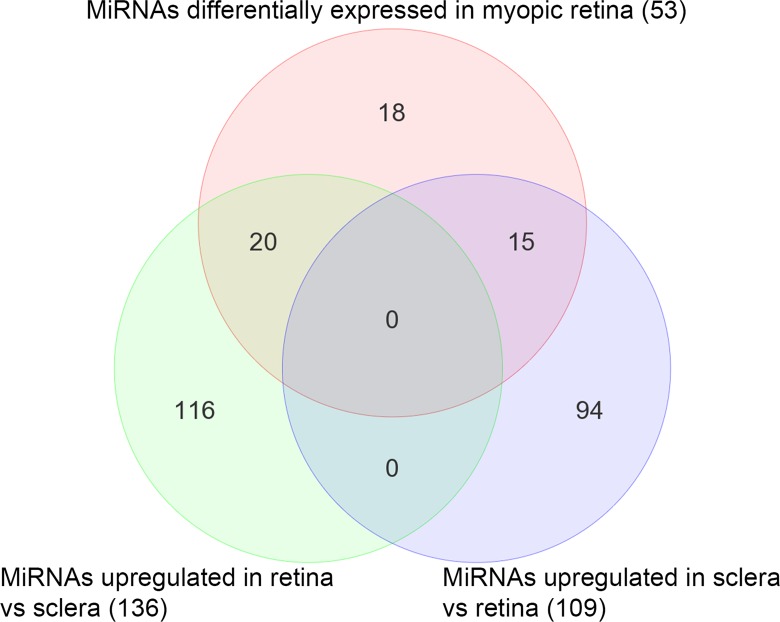
Overlap between miRNAs differentially expressed in the myopic retina and miRNAs differentially expressed in the retina versus sclera. Venn diagram shows overlap between 53 miRNAs, which were differentially expressed in the myopic retina, 136 miRNAs, which were up-regulated in the retina versus sclera, and 109 miRNAs, which were up-regulated in the sclera versus retina. Eighteen differential miRNAs were equally expressed in both retina and sclera, 20 differential miRNAs were up-regulated in the retina versus sclera and 15 differential miRNAs were down-regulated in the retina versus sclera.

To examine potential involvement of miRNAs in the development of myopia, we analyzed miRNA expression in a mouse model of myopia. Twelve P24 C57BL/6J mice were subjected to monocular visual form deprivation (VFD). After 10 days of VFD, we detected a myopic shift in refraction in the deprived eyes of -6.93 ± 2.44 D (p < 0.000001, n = 12) relative to the control eyes (Fig 1). The difference in the interocular difference (OD-OS) between the VFD group and control group was also statistically significant (F(1, 18) = 49.936, p < 0.000001). A large-scale miRNA expression profiling was performed in the retina and sclera of the VFD mice using Agilent mouse microRNA microarrays which contained 627 mature mouse and 39 mouse viral miRNAs. This profiling revealed that a total of 53 miRNAs were differentially expressed (FC ≥ 2.0; FDR-adjusted p < 0.05) in the myopic retina compared to the contralateral control retina, whereas no differentially expressed miRNAs were identified in the sclera (Fig 2; Table 1). Thirty seven out of the 53 miRNAs were up-regulated and 16 out of the 53 miRNAs were down-regulated in the myopic retina (Fig 2; Table 1). Analysis of differential expression of these 53 miRNAs in the retina versus sclera revealed that 18 miRNAs were equally expressed in both retina and sclera, whereas 20 miRNAs were upregulated in the retina versus sclera and 15 were upregulated in the sclera versus retina (Fig 3; S1 Fig; S1 Table). Although the majority of the differentially expressed miRNAs originated from different miRNA clusters, mmu-miR-429-3p and mmu-miR-200a-5p belonged to the same cluster (MID < 5 kb) on chromosome 4 and were both up-regulated in myopic retina, while mmu-miR-145-5p and mmu-miR-143-3p localized within the same cluster (MID < 5 kb) on chromosome 18 and were both down-regulated in myopic retina (Table 1) [68]. Several miRNAs exhibited more than 10-fold change in expression in the myopic retina (Table 1), including mmu-miR-1947-5p (FC = 31.5, p = 1.47 × 10^−04^), mmu-miR-200a-5p (FC = 18.8, p = 9.46 × 10^−05^), mmu-miR-141-5p (FC = 13.9, p = 4.75 × 10^−06^), mmu-miR-465b-5p (FC = 12.8, p = 5.93 × 10^−04^), mmu-miR-214-5p (FC = 12.6, p = 8.27 × 10^−03^), mmu-miR-1936 (FC = 12.3, p = 9.56 × 10^−06^), mmu-miR-466f-5p (FC = 11.5, p = 3.85 × 10^−03^), mmu-miR-669o-5p (FC = 10.9, p = 2.18 × 10^−03^), mmu-miR-18b-5p (FC = 10.1, p = 1.79 × 10^−03^), and mmu-miR-145-5p (FC = -10.5, p = 8.87 × 10^−09^). These data suggest that development of form-deprivation myopia is associated with large-scale changes in miRNA expression in the retina.

**Table 1:** MiRNAs differentially expressed in myopic retina versus control retina.

miRBase ID	miRNA name	miRNA cluster	Fold change	P-value
MIMAT0009413	mmu-miR-1947-5p	miR-1947-5p (miRNAs w/seed GGACGAG)	31.5	1.47 × 10^−04^
MIMAT0004619	mmu-miR-200a-5p	miR-200a-5p (and other miRNAs w/seed AUCUUAC)	18.8	9.46 × 10^−05^
MIMAT0004533	mmu-miR-141-5p	miR-141-5p (and other miRNAs w/seed AUCUUCC)	13.9	4.75 × 10^−06^
MIMAT0004871	mmu-miR-465b-5p	miR-465b-5p (and other miRNAs w/seed AUUUAGA)	12.8	5.93 × 10^−04^
MIMAT0004664	mmu-miR-214-5p	miR-214-5p (miRNAs w/seed GCCUGUC)	12.6	8.27 × 10^−03^
MIMAT0009400	mmu-miR-1936	miR-1936 (miRNAs w/seed AACUGAC)	12.3	9.56 × 10^−06^
MIMAT0004881	mmu-miR-466f-5p	miR-466f-5p (miRNAs w/seed ACGUGUG)	11.5	3.85 × 10^−03^
MIMAT0009421	mmu-miR-669o-5p	miR-669o-5p (miRNAs w/seed AGUUGUG)	10.9	2.18 × 10^−03^
MIMAT0004858	mmu-miR-18b-5p	miR-18a-5p (and other miRNAs w/seed AAGGUGC)	10.1	1.79 × 10^−03^
MIMAT0009411	mmu-miR-1306-3p	miR-1306-3p (miRNAs w/seed CGUUGGC)	9.3	3.67 × 10^−03^
MIMAT0000368	mmu-miR-291a-3p	miR-291a-3p (and other miRNAs w/seed AAGUGCU)	9.2	3.22 × 10^−04^
MIMAT0003483	mmu-miR-696	miR-696 (miRNAs w/seed CGUGUGC)	9.0	1.28 × 10^−03^
MIMAT0004628	mmu-miR-21-3p	miR-21-3p (and other miRNAs w/seed AACAGCA)	8.9	8.01 × 10^−05^
MIMAT0004824	mmu-miR-673-3p	miR-673-3p (and other miRNAs w/seed CCGGGGC)	8.4	1.65 × 10^−05^
MIMAT0001537	mmu-miR-429-3p	miR-200b-3p (and other miRNAs w/seed AAUACUG)	7.8	2.05 × 10^−03^
MIMAT0011212	mmu-miR-2136	miR-2136 (miRNAs w/seed UGGGUGU)	6.3	5.02 × 10^−04^
MIMAT0004841	mmu-miR-871-5p	miR-743a-5p (and other miRNAs w/seed AUUCAGA)	5.8	5.51 × 10^−04^
MIMAT0004526	mmu-miR-101a-5p	miR-101a-5p (miRNAs w/seed CAGUUAU)	5.1	1.88 × 10^−04^
MIMAT0007873	mmu-miR-1896	miR-1896 (miRNAs w/seed UCUCUGA)	4.9	1.66 × 10^−03^
MIMAT0004885	mmu-miR-467c-5p	miR-467c-5p (and other miRNAs w/seed AAGUGCG)	4.6	1.68 × 10^−04^
MIMAT0004884	mmu-miR-466h-5p	miR-669m-5p (and other miRNAs w/seed GUGUGCA)	4.3	1.29 × 10^−03^
MIMAT0003476	mmu-miR-669b-5p	miR-669b-5p (miRNAs w/seed GUUUUGU)	4.3	1.02 × 10^−03^
MIMAT0005853	mmu-miR-669e-5p	miR-331-5p (and other miRNAs w/seed GUCUUGU)	4.2	2.41 × 10^−03^
MIMAT0004856	mmu-miR-105	miR-105 (miRNAs w/seed CAAGUGC)	4.2	1.83 × 10^−04^
MIMAT0003494	mmu-miR-704	miR-704 (miRNAs w/seed GACAUGU)	4.0	3.00 × 10^−04^
MIMAT0000372	mmu-miR-294-3p	miR-291a-3p (and other miRNAs w/seed AAGUGCU)	3.9	3.79 × 10^−03^
MIMAT0004626	mmu-miR-18a-3p	miR-18a-3p (and other miRNAs w/seed CUGCCCU)	3.9	6.01 × 10^−04^
MIMAT0003169	mmu-miR-539-5p	miR-539-5p (miRNAs w/seed GAGAAAU)	3.5	3.47 × 10^−04^
MIMAT0009427	mmu-miR-669n	miR-5010-3p (and other miRNAs w/seed UUUGUGU)	3.3	7.22 × 10^−03^
MIMAT0004647	mmu-miR-338-5p	miR-338-5p (miRNAs w/seed ACAAUAU)	3.2	4.09 × 10^−04^
MIMAT0003460	mmu-miR-449c-5p	miR-34a-5p (and other miRNAs w/seed GGCAGUG)	2.6	1.41 × 10^−04^
MIMAT0000239	mmu-miR-206-3p	miR-1-3p (and other miRNAs w/seed GGAAUGU)	2.5	4.14 × 10^−04^
MIMAT0007868	mmu-miR-1903	miR-1903 (and other miRNAs w/seed CUUCUUC)	2.4	5.95 × 10^−04^
MIMAT0000380	mmu-miR-302a-3p	miR-291a-3p (and other miRNAs w/seed AAGUGCU)	2.4	5.27 × 10^−04^
MIMAT0014816	mmu-miR-3099-3p	miR-3099 (and other miRNAs w/seed AGGCUAG)	2.4	7.47 × 10^−03^
MIMAT0003507	mmu-miR-500-3p	miR-501-3p (and other miRNAs w/seed AUGCACC)	2.2	4.14 × 10^−03^
MIMAT0000246	mmu-miR-122-5p	miR-122-5p (miRNAs w/seed GGAGUGU)	2.0	8.09 × 10^−04^
MIMAT0000247	mmu-miR-143-3p	miR-143-3p (and other miRNAs w/seed GAGAUGA)	-2.0	1.43 × 10^−03^
MIMAT0003738	mmu-miR-496-3p	miR-503-3p (and other miRNAs w/seed GAGUAUU)	-2.1	8.01 × 10^−10^
MIMAT0001418	mmu-miR-431-5p	miR-431-5p (and other miRNAs w/seed GUCUUGC)	-2.1	7.76 × 10^−04^
MIMAT0000137	mmu-miR-126-5p	miR-126a-5p (and other miRNAs w/seed AUUAUUA)	-2.3	3.67 × 10^−03^
MIMAT0004572	mmu-miR-290-3p	miR-467a-5p (and other miRNAs w/seed AAGUGCC)	-2.4	6.43 × 10^−03^
MIMAT0003731	mmu-miR-671-5p	miR-671-5p (miRNAs w/seed GGAAGCC)	-2.6	2.30 × 10^−03^
MIMAT0003729	mmu-miR-216b-5p	miR-216b-5p (miRNAs w/seed AAUCUCU)	-2.6	1.51 × 10^−05^
MIMAT0000665	mmu-miR-223-3p	miR-223-3p (miRNAs w/seed GUCAGUU)	-2.7	4.84 × 10^−04^
MIMAT0003782	mmu-miR-676-3p	miR-676 (and other miRNAs w/seed CGUCCUG)	-2.7	9.35 × 10^−04^
MIMAT0004640	mmu-miR-325-3p	miR-325-3p (miRNAs w/seed UUAUUGA)	-2.8	5.59 × 10^−04^
MIMAT0003742	mmu-miR-455-3p	miR-455-3p (miRNAs w/seed CAGUCCA)	-3.1	1.74 × 10^−03^
MIMAT0000229	mmu-miR-199a-5p	miR-199a-5p (and other miRNAs w/seed CCAGUGU)	-3.2	6.65 × 10^−03^
MIMAT0000158	mmu-miR-146a-5p	miR-146a-5p (and other miRNAs w/seed GAGAACU)	-3.2	2.70 × 10^−05^
MIMAT0000155	mmu-miR-142-3p	miR-142-3p (and other miRNAs w/seed GUAGUGU)	-3.3	2.13 × 10^−03^
MIMAT0004528	mmu-miR-125a-3p	miR-125a-3p (miRNAs w/seed CAGGUGA)	-3.4	1.21 × 10^−03^
MIMAT0000157	mmu-miR-145-5p	miR-145-5p (and other miRNAs w/seed UCCAGUU)	-10.5	8.87 × 10^−09^

### Identification of putative target mRNAs for the miRNAs differentially expressed in myopic retina

**Fig 4:**
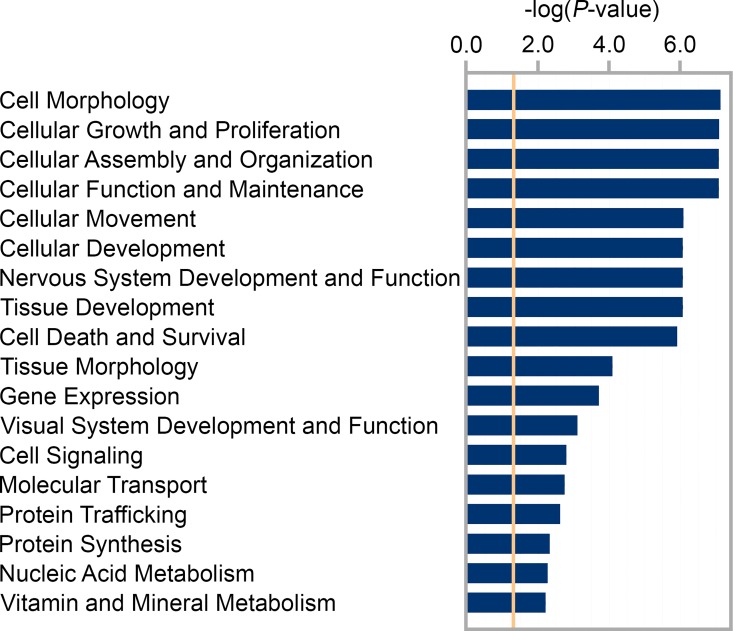
Gene ontology categories affected in myopic retina. Graph shows top 18 biological processes which were modified in the myopic retina.

In order to explore potential signaling pathways regulated by the identified differentially expressed miRNAs during the development of form-deprivation myopia in mice, we performed miRNA-mRNA interaction network analyses using a custom database of mRNAs differentially expressed in the retina upon induction of experimental myopia (S2 Table) [10, 13, 14, 61, 62] using IPA^®^. This analysis identified a total of 135 mRNA targets for 21 out of 53 differentially expressed miRNAs (Table 2), whereas no target mRNAs were found for the remaining 32 miRNAs among 611 mRNAs, which were found to be differentially expressed in the myopic retina (S3 Table). Gene ontology analysis of the target mRNAs revealed that these mRNAs encode proteins primarily involved in cellular growth, proliferation, nervous and visual systems development (Fig 4). The miRNA-mRNA networks within these gene ontology categories were characterized by complex combinatorial interactions with one miRNA targeting multiple mRNAs and one mRNA often being targeted by several miRNAs. For example, mmu-miR-145-5p, which was strongly down-regulated in myopic retina (FC = -10.5, p = 8.87 × 10^−09^), targeted 25 mRNAs (the largest number among all 21 miRNAs) (Table 2); while mmu-miR-429-3p (FC = 7.8, p = 2.05 × 10^−03^), mmu-miR-143-3p (FC = -2.0, p = 1.43 × 10^−03^), mmu-miR-223-3p (FC = -2.7, p = 4.84 × 10^−04^) and mmu-miR-146a-5p (FC = -3.2, p = 2.70 × 10^−05^) targeted 17, 17, 16 and 14 mRNAs respectively. The average number of mRNAs targeted by one miRNA was 9.0 ± 6.2. On the other hand, each mRNA was targeted by only 1.4 ± 0.7 miRNAs and maximum by 4 miRNAs.

**Table 2:** MiRNAs and their target mRNAs differentially expressed in myopic retina versus control retina.

miRNA name	miRNA fold change	Target mRNA name and fold change
mmu-miR-18b-5p	10.1	BRWD3 (-2.5); NOS1 (-2.0); CTGF (-2.0); NXT2 (-1.7); **HLF** (-1.6); TMEM2 (-1.6)
mmu-miR-1306-3p	9.3	DUSP4 (-2.5)
mmu-miR-291a-3p	9.2	**CNOT6** (-2.2); RAB11A (-2.0); CUL3 (-1.7); CLIP4 (-1.6); **HLF** (-1.6); BAZ1A (-1.6); ZC3H13 (-1.2); **ZFP91** (-1.0)
mmu-miR-429-3p	7.8	**ZEB2** (-3.5); ATP11B (-2.7); PRKAR1A (-2.6); BRWD3 (-2.5); RIMS2 (-1.9); **RBFOX2** (-1.8); SENP5 (-1.8); ARIH1 (-1.7); MAPK9 (-1.7); **COMMD3** (-1.7); **HLF** (-1.6); SLC30A5 (-1.6); TAOK3 (-1.6); **ETV5** (-1.5); NEGR1 (-1.5); MPRIP (-1.2); MAP2 (-1.2)
mmu-miR-539-5p	3.5	RAB11A (-2.0); SENP5 (-1.8); WNK1 (-1.8); NPL (-1.7); ZSWIM5 (-1.7); MAP2 (-1.2)
mmu-miR-449c-5p	2.6	NOS1 (-2.0); **RBFOX2** (-1.8); MRPL17 (-1.7); COPS7B (-1.7); ZSWIM5 (-1.7); **TCF12** (-1.6)
mmu-miR-206-3p	2.5	**CNOT6** (-2.2); MAN1C1 (-1.8); ARIH1 (-1.7); NXT2 (-1.7); RICTOR (-1.7); TAOK3 (-1.6); FAM101B (-1.6); KLHL5 (-1.5); **ZFP91** (-1.0)
mmu-miR-1903	2.4	**ASCL1** (-1.6)
mmu-miR-500-3p	2.2	OLFM4 (-3.5)
mmu-miR-122-5p	2.0	DUSP4 (-2.5); **UBAP2** (-1.9); NEGR1 (-1.5)
mmu-miR-143-3p	-2.0	FUT4 (1.2); COX18 (1.2); CHST10 (1.2); BCL2 (1.2); PPP2R3A (1.3); GABARAPL1 (1.3); DCLK1 (1.3); HTR7 (1.3); **LBH** (1.3); GXYLT1 (1.3); ADD3 (1.5); **ZNF275** (1.5); MAP1B (1.6); DCX (1.6); PRKCE (1.8); RNF165 (1.8); TPM3 (1.9)
mmu-miR-496-3p	-2.1	**CSRNP3** (1.2); CNOT2 (1.2); **AEBP2** (1.2); INSIG1 (1.3); PRKCE (1.8); NT5C2 (6.3)
mmu-miR-431-5p	-2.1	PTPRF (1.1); AKAP12 (1.3); ZAK (1.6); THUMPD1 (1.6); CELF2 (1.8)
mmu-miR-671-5p	-2.6	MPEG1 (1.0); ANKS1A (1.2); RBMS3 (1.2); **NFYA** (1.3); MYOM3 (1.4); KIAA0430 (1.5); ATXN7L1 (1.6); SEPP1 (1.7); SAMD12 (1.8); SPTBN1 (2.9)
mmu-miR-216b-5p	-2.6	**E2F4** (1.1); NRK (1.2); MCM4 (1.2); **NFYA** (1.3); FBXO8 (1.3); CHMP1B (1.5); GLTSCR1L (1.5); SDHC (1.6); SAR1B (1.6); CELF2 (1.8); TPM3 (1.9); SPTBN1 (2.9); GFRA1 (3.8)
mmu-miR-223-3p	-2.7	**NUCKS1** (1.1); EBNA1BP2 (1.2); PARP1 (1.2); CNOT2 (1.2); ATG7 (1.2); **AEBP2** (1.2); SYNCRIP (1.3); DDIT4 (1.3); FBXO8 (1.3); NUP210 (1.4); ARFIP1 (1.6); ATXN7L1 (1.6); **WDR77** (1.6); PRR14L (1.8); PRKCE (1.8); ERC1 (4.8)
mmu-miR-199a-5p	-3.2	**TOX3** (1.3); **NFYA** (1.3); MCFD2 (1.3); CCNJ (1.3); NUP210 (1.4); ARHGEF12 (1.5); ADD3 (1.5); RALGAPA1 (1.5); ATXN7L1 (1.6); CELF2 (1.8)
mmu-miR-146a-5p	-3.2	CAMSAP1 (1.1); **NUCKS1** (1.1); CCNA2 (1.2); LFNG (1.2); ATG7 (1.2); CCNJ (1.3); NAIF1 (1.3); EDNRB (1.5); NLGN1 (1.6); CELF2 (1.8); PRKCE (1.8); **NOTCH2** (2.0); SBSPON (2.0); **STAT1** (2.7)
mmu-miR-142-3p	-3.3	**NUCKS1** (1.1); ANKS1A (1.2); HGS (1.2); **MORF4L2** (1.2); ZCCHC24 (1.3); CCNJ (1.3); ARHGEF12 (1.5); MYLK (1.7); SAMD12 (1.8); ERC1 (4.8)
mmu-miR-125a-3p	-3.4	MAPK1IP1L (1.2); **AEBP2** (1.2); SWAP70 (1.4); FYCO1 (1.8); RAB22A (2.2)
mmu-miR-145-5p	-10.5	CCNA2 (1.2); SMC1A (1.2); DENND4B (1.2); KIAA0930 (1.2); KATNBL1 (1.2); PPP2R3A (1.3); GABARAPL1 (1.3); **HABP4** (1.3); PTGR2 (1.3); GCLM (1.3); AKAP12 (1.3); GXYLT1 (1.3); SWAP70 (1.4); VSTM4 (1.4); ARHGEF12 (1.5); ADD3 (1.5); PHACTR2 (1.6); DOK6 (1.6); ATXN7L1 (1.6); QSER1 (1.8); **COMMD5** (1.9); TPM3 (1.9); ANKRD28 (1.9); ANGPT2 (2.7); GFRA1 (3.8)

Transcription factors are shown in bold; genes involved in synapse formation or function are underlined.

### Analysis of miRNA-regulated signaling pathways in the mouse form-deprivation myopia

**Fig 5:**
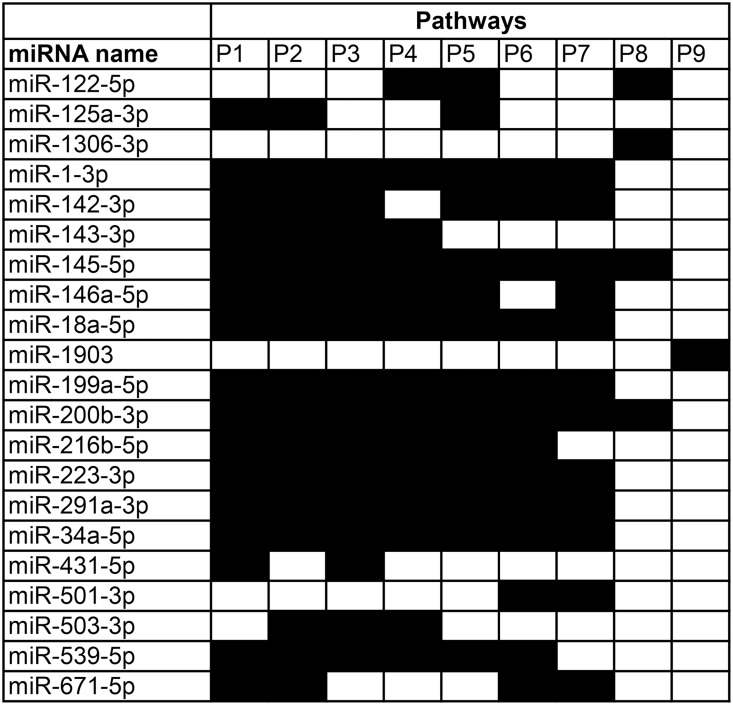
Overlap between miRNA-regulated signaling pathways affected in myopic retina. Diagram depicts miRNA contributions to the 9 miRNA-mRNA signaling cascades associated with form-deprivation myopia in mice.

Our initial analysis revealed that each differentially expressed miRNA regulates an extended network of protein-coding genes within a limited number of gene ontology categories, therefore we then explored interactions between 21 differentially expressed miRNAs and 135 mRNAs which were targeted by these miRNAs. This analysis revealed that these miRNAs and mRNAs are organized into 9 overlapping miRNA-mRNA signaling pathways (Fig 5; S2–S10 Figs). Myopia pathways (MP) 1 and 2 (MP1 and MP2) were the largest pathways, which comprised 16 miRNAs, followed by MP3 (15 miRNAs), MP4 (14 miRNAs), MP5 (14 miRNAs), MP6 (13 miRNAs), MP7 (12 miRNAs), MP8 (4 miRNAs) and MP9 (1 miRNA). MP1, MP2, MP3, MP4, MP5, MP6 and MP7 had a common core comprised of mmu-miR-1-3p, mmu-miR-145-5p, mmu-miR-18a-5p, mmu-miR-199a-5p, mmu-miR-200b-3p, mmu-miR-223-3p, mmu-miR-291a-3p, mmu-miR-34a-5p and their target mRNAs. Two of these miRNAs, i.e., mmu-miR-145-5p and mmu-miR-200b-3p, served as a common core for all pathways except for MP9. MP9 comprised only 1 miRNA (mmu-miR-1903) and 3 mRNAs (Ascl1, Dcx and Notch2), but was linked to 6 larger pathways, i.e., MP1, MP2, MP3, MP4, MP5 and MP7, via Notch2 (indirect target of mmu-miR-1903). In all pathways, miRNAs played a role of major regulatory hubs targeting transcription factors and/or regulatory proteins. The absolute majority of miRNAs targeted at least one transcription factor while the average number of transcription factors targeted by each differentially expressed miRNA was 1.7 ± 1.2 (Table 2). At the same time, several transcription factors were targeted by multiple often overlapping miRNAs. For example, Hlf was targeted by mmu-miR-18b-5p, mmu-miR-429-3p and mmu-miR-291a-3p; Cnot6 and Zfp91 were targeted by mmu-miR-206-3p and mmu-miR-291a-3p; Rbfox2 was targeted by mmu-miR-429-3p and mmu-miR-449c-5p; Aebp2 was targeted by mmu-miR-125a-3p, mmu-miR-223-3p and mmu-miR-496-3p; Nfya was targeted by mmu-miR-199a-5p, mmu-miR-216b-5p and mmu-miR-671-5p; Nucks1 was targeted by mmu-miR-142-3p, mmu-miR-146a-5p and mmu-miR-223-3p; Notch2 was targeted by mmu-miR-146a-5p and indirectly via Ascl1 by mmu-miR-1903. Interestingly, mmu-miR-145-5p and mmu-miR-429-3p (mmu-miR-200b-3p cluster), which formed a common core of all pathways, were among the most differential miRNAs and targeted the largest number of mRNAs. Mmu-miR-145-5p was strongly down-regulated in myopic retina (FC = -10.5, p = 8.87 × 10^−09^) and targeted 25 mRNAs, while mmu-miR-429-3p was strongly up-regulated in myopic retina (FC = 7.8, p = 2.05 × 10^−03^) and targeted 17 mRNAs (Table 2).

Among the mRNAs targeted by differentially expressed miRNAs, there were several (Prkar1a, Rims2, Map2, Gabarapl1, Htr7, Add3, Erc1, Nlgn1) which were involved in synapse formation and function suggesting that synaptic function was one of the biological processes affected in form-deprivation myopia. However, IPA gene ontology analysis revealed that the most prominent biological processes associated with the 21 differentially expressed miRNAs and their target mRNAs were quantity of neurons (p = 1.08 × 10^−4^, activation z-score (z) = 2.04), migration of neurons (p = 2.24 × 10^−6^, z = 1.33), growth of axons (p = 7.21 × 10^−4^, z = 1.10), outgrowth of neurites (p = 1.83 × 10^−5^, z = 0.74), growth of neurites (p = 8.51 × 10^−7^, z = 0.54), neuritogenesis (p = 1.03 × 10^−6^, z = 0.41), proliferation of neuronal cells (p = 9.93 × 10^−7^, z = 0.33) and differentiation of neurons (p = 1.45 × 10^−3^, z = 0.17) (S4 Table). Therefore, we have analyzed the overlap between all 9 pathways and GO categories linked to neurogenesis. We found that each pathway was indeed involved in regulation of neurogenesis and had a net positive effect on neurogenesis-associated processes (S2–S10 Figs). Thus, pathway analysis suggests that miRNAs serve as key regulators of several signaling cascades underlying development of form-deprivation myopia.

## Discussion

Development of myopia is associated with remodeling of several ocular tissues, including the retina, RPE, choroid and sclera [14, 69–72]. Several studies analyzed gene expression in various animal models of myopia using oligonucleotide-based microarrays and each identified between 15 and 280 differentially expressed genes [10–14, 61, 62], including a total of 611 mRNAs in the retina [10, 13, 14, 61, 62]. Although involvement of coding genes in myopia is a well-established fact, to date only 1 miRNA was shown to be associated with myopia development. It was reported that a SNP located within the miR-328 binding site in the 3’-UTR of *PAX6* reduced *PAX6* protein levels and was significantly associated with extreme myopia in a Chinese cohort [54, 55]. Considering that miRNAs often serve as regulatory hubs in many signaling pathways [21–24] and provide higher-order coordination of signaling pathways underlying the same biological process [68, 73], understanding the role of miRNAs in refractive eye development is critical for the understanding of myopia development.

In this work, we sought to identify miRNAs differentially expressed during the development of form-deprivation myopia in the mouse model and to explore miRNA-mRNA genetic networks underlying myopia. Considering that mice are most susceptible to visually-guided myopia during active phase of ocular growth from P21 through P40 [60, 74], we analyzed early changes in miRNA expression in the retina and sclera in P34 mice 10 days after induction of form-deprivation myopia. Ten days of visual form deprivation induced -6.93 ± 2.44 diopters of myopia and large-scale changes in miRNA expression in the retina indicating that early retinal response in visually-guided myopia is associated with substantial changes in retinal signaling. Surprisingly, we did not find differences in miRNA expression in the sclera, which suggests that early myopia-associated scleral remodeling in response to visual form deprivation may not involve miRNAs, or that changes in miRNA expression are very subtle at this early stage of myopia development. In the retina, we identified 53 differentially expressed miRNAs, including 19 miRNAs with more than 5-fold change in expression. Importantly, 18 of these highly differential miRNAs were upregulated in the myopic retina and only 1 was down-regulated suggesting that the “net effect” may be the down-regulation of the corresponding target genes in myopia. Seven of the up-regulated miRNAs (miR-465b, miR-466f, miR-669o, miR-18b, miR-291a, miR-696, miR-101a) were also much more (≥ 5 fold) expressed in the retina compared to the sclera suggesting that they might be involved in the regulation of retina-specific processes. The down-regulated miR-145 was 25.4 times less abundant in the retina versus sclera suggesting that it is likely to be expressed in a very small population of retinal cells. Several of the highly differential miRNAs were previously shown to be key regulators of various developmental processes. For example, miR-200a, miR-429 and miR-141 were shown to play important roles in neurogenesis, epithelial-to-mesenchymal transition and Notch signaling [73, 75–84], miR-214 was found to be overexpressed in fetal sclera versus adult sclera and shown to play important role in brain and retina development and function [36, 85–89], miR-18b, miR-21, miR-101a, miR-200a and miR-429 were found to be involved in stem cell function and differentiation [90–100], miR-1306 negatively regulated Alzheimer’s disease gene ADAM10 [101]. MicroRNA miR-145 regulates stem cell, smooth muscle cell, corneal epithelium and mesenchymal stem cell differentiation [102–107], as well as intestine and neural crest development [108, 109]. MiR-145 was also shown to regulate L-DOPA decarboxylase [110], which is one of the key enzymes synthesizing dopamine in the dopaminergic amacrine cells in the retina [111]. Thus, differential expression of these microRNAs suggests that form-deprivation myopia is associated with changes in neurogenesis, as well as in neuronal and synaptic functions.

Analysis of the mRNAs targeted by the differentially expressed miRNAs revealed that putative mRNA targets could be found for 21 out of 53 differential miRNAs (i.e., for 40% of all differential miRNAs), suggesting that the currently available list of mRNAs differentially expressed in the myopic retina is not complete and many retinal mRNAs underlying myopia are still unknown. However, the 21 differential miRNAs and their associated targets provided a sufficient foundation for the analysis of main biological processes and pathways associated with myopia development. Gene ontology analysis revealed that such biological processes as generation of new neurons, migration of neurons, growth of axons, outgrowth of neurites, cellular growth and proliferation, nervous and visual systems development, which were suggested to be affected based on the analysis of the most differential miRNAs, were significantly enhanced in myopic retina. Interestingly, this finding is consistent with the recent report that form-deprivation myopia in primates is associated with increased proliferation of retinal progenitor cells and retinal growth [14], thus, providing an outline of the putative molecular network underlying retinal growth associated with myopia development. Several target mRNAs were also linked to synaptic structure and function, which is consistent with the observations in animal models that synaptic signaling at the level of amacrine cells is involved in myopia development [112–123]. Remarkably, analysis of the miRNA-mRNA networks formed by the differential miRNAs and their targets revealed that myopia development is, in fact, regulated by a small number of highly integrated signaling pathways. MicroRNAs played a role of master regulators, which targeted large number of mRNAs often involved in the same biological process. Furthermore, miRNAs often had common transcription factors among the targets, which provides an additional level of integration. Interestingly, two miRNAs miR-145 and miR-200b seemed to play a role of the integrative core for all pathways.

Taken together, our findings suggest that the miRNAs differentially expressed in the retina of myopic eyes play important regulatory roles in the development of myopia by regulating a highly integrated genetic network. We analyzed expression of 56% of mouse miRNAs deposited in the miRBase database and identified 53 miRNAs differentially expressed in the retina during development of form-deprivation myopia. We also identified 135 target genes for 21 of these miRNAs and reconstructed putative miRNA-mRNA pathways underlying key biological processes associated with development of form-deprivation myopia. These results expand our understanding of the molecular mechanisms of myopia and demonstrate that the development of myopia is associated with large-scale changes in expression of both coding and none-coding RNAs. The power of our analysis was limited by the cellular complexity of the retina as well as some differences in cell composition of the retina in different species. Although it was shown that processing of defocus and refractive eye development are regulated by a relatively small subset of retinal cells (i.e., amacrine cells) [112–123], detecting small changes in gene expression might be challenging in heterogeneous tissues such as retina, resulting in an underestimate of miRNA influences. It is also a challenge to place differential miRNAs and their corresponding target mRNAs in the proper cellular context when analyzing gene expression in complex tissues. Future studies of the signaling pathways underlying myopia development at the single-cell level should provide more accurate information. Our data also suggest that more comprehensive genome-wide approaches should be applied to reconstruct signaling pathways underlying myopia in their entirety. This study lays a strong foundation for such future studies and provides a framework for the development of potential novel microRNA-based therapies for myopia.

## Supporting Information

S1 Fig Hierarchical cluster analysis of 245 miRNAs differentially expressed in the retina versus sclera. Logarithmic values (base 2) of Agilent total gene signal for differentially expressed miRNAs (cutoff: FC > 2, FDR-adjusted p-value < 0.05) were quantile normalized, shifted to mean zero, scaled to standard deviation of 1.0 and subjected to hierarchical clustering using Euclidean dissimilarity and average linkage. The color scale indicates transcript abundance: red identifies an increase in relative miRNA abundance; blue identifies a decrease in relative miRNA abundance. Columns show individual samples, whereas rows show individual miRNAs.
(TIF)

S2 Fig MiRNA-mRNA signaling pathway #1. (A) Diagram showing interactions between miRNAs differentially expressed in myopic retina and their target mRNAs. Arrows show relationships between different miRNAs and mRNAs. (B) Overlap between signaling pathway #1 and top gene ontology categories. Red identifies genes/categories which are up-regulated in myopic retina, whereas green identifies genes/categories which are down-regulated in myopic retina.
(TIF)

S3 Fig MiRNA-mRNA signaling pathway #2. (A) Diagram showing interactions between miRNAs differentially expressed in myopic retina and their target mRNAs. Arrows show relationships between different miRNAs and mRNAs. (B) Overlap between signaling pathway #2 and top gene ontology categories. Red identifies genes/categories which are up-regulated in myopic retina, whereas green identifies genes/categories which are down-regulated in myopic retina.
(TIF)

S4 Fig MiRNA-mRNA signaling pathway #3. (A) Diagram showing interactions between miRNAs differentially expressed in myopic retina and their target mRNAs. Arrows show relationships between different miRNAs and mRNAs. (B) Overlap between signaling pathway #3 and top gene ontology categories. Red identifies genes/categories which are up-regulated in myopic retina, whereas green identifies genes/categories which are down-regulated in myopic retina.
(TIF)

S5 Fig MiRNA-mRNA signaling pathway #4. (A) Diagram showing interactions between miRNAs differentially expressed in myopic retina and their target mRNAs. Arrows show relationships between different miRNAs and mRNAs. (B) Overlap between signaling pathway #4 and top gene ontology categories. Red identifies genes/categories which are up-regulated in myopic retina, whereas green identifies genes/categories which are down-regulated in myopic retina.
(TIF)

S6 Fig MiRNA-mRNA signaling pathway #5. (A) Diagram showing interactions between miRNAs differentially expressed in myopic retina and their target mRNAs. Arrows show relationships between different miRNAs and mRNAs. (B) Overlap between signaling pathway #5 and top gene ontology categories. Red identifies genes/categories which are up-regulated in myopic retina, whereas green identifies genes/categories which are down-regulated in myopic retina.
(TIF)

S7 Fig MiRNA-mRNA signaling pathway #6. (A) Diagram showing interactions between miRNAs differentially expressed in myopic retina and their target mRNAs. Arrows show relationships between different miRNAs and mRNAs. (B) Overlap between signaling pathway #6 and top gene ontology categories. Red identifies genes/categories which are up-regulated in myopic retina, whereas green identifies genes/categories which are down-regulated in myopic retina.
(TIF)

S8 Fig MiRNA-mRNA signaling pathway #7. (A) Diagram showing interactions between miRNAs differentially expressed in myopic retina and their target mRNAs. Arrows show relationships between different miRNAs and mRNAs. (B) Overlap between signaling pathway #7 and top gene ontology categories. Red identifies genes/categories which are up-regulated in myopic retina, whereas green identifies genes/categories which are down-regulated in myopic retina.
(TIF)

S9 Fig MiRNA-mRNA signaling pathway #8. (A) Diagram showing interactions between miRNAs differentially expressed in myopic retina and their target mRNAs. Arrows show relationships between different miRNAs and mRNAs. (B) Overlap between signaling pathway #8 and top gene ontology categories. Red identifies genes/categories which are up-regulated in myopic retina, whereas green identifies genes/categories which are down-regulated in myopic retina.
(TIF)

S10 Fig MiRNA-mRNA signaling pathway #9. (A) Diagram showing interactions between miRNAs differentially expressed in myopic retina and their target mRNAs. Arrows show relationships between different miRNAs and mRNAs. (B) Overlap between signaling pathway #9 and top gene ontology categories. Red identifies genes/categories which are up-regulated in myopic retina, whereas green identifies genes/categories which are down-regulated in myopic retina.
(TIF)

S1 Table MiRNAs differentially expressed in myopic retina versus control retina and their expression between retina and sclera. (XLSX)

S2 Table Messenger RNAs differentially expressed in myopic retina. (XLSX)

S3 Table MiRNAs without target mRNAs differentially expressed in myopic retina versus control retina. (DOCX)

S4 Table Top gene ontology categories associated with 21 miRNAs differentially expressed in the mouse form-deprivation myopia. (XLSX)
