# Supplementary figures and images for: Large-Scale microRNA Expression Profiling Identifies Putative Retinal miRNA-mRNA Signaling Pathways Underlying Form-Deprivation Myopia in Mice
Source: PLoS One. 2016 Sep 13;11(9):e0162541. doi: 10.1371/journal.pone.0162541 (PMC5021328; doi:10.1371/journal.pone.0162541)

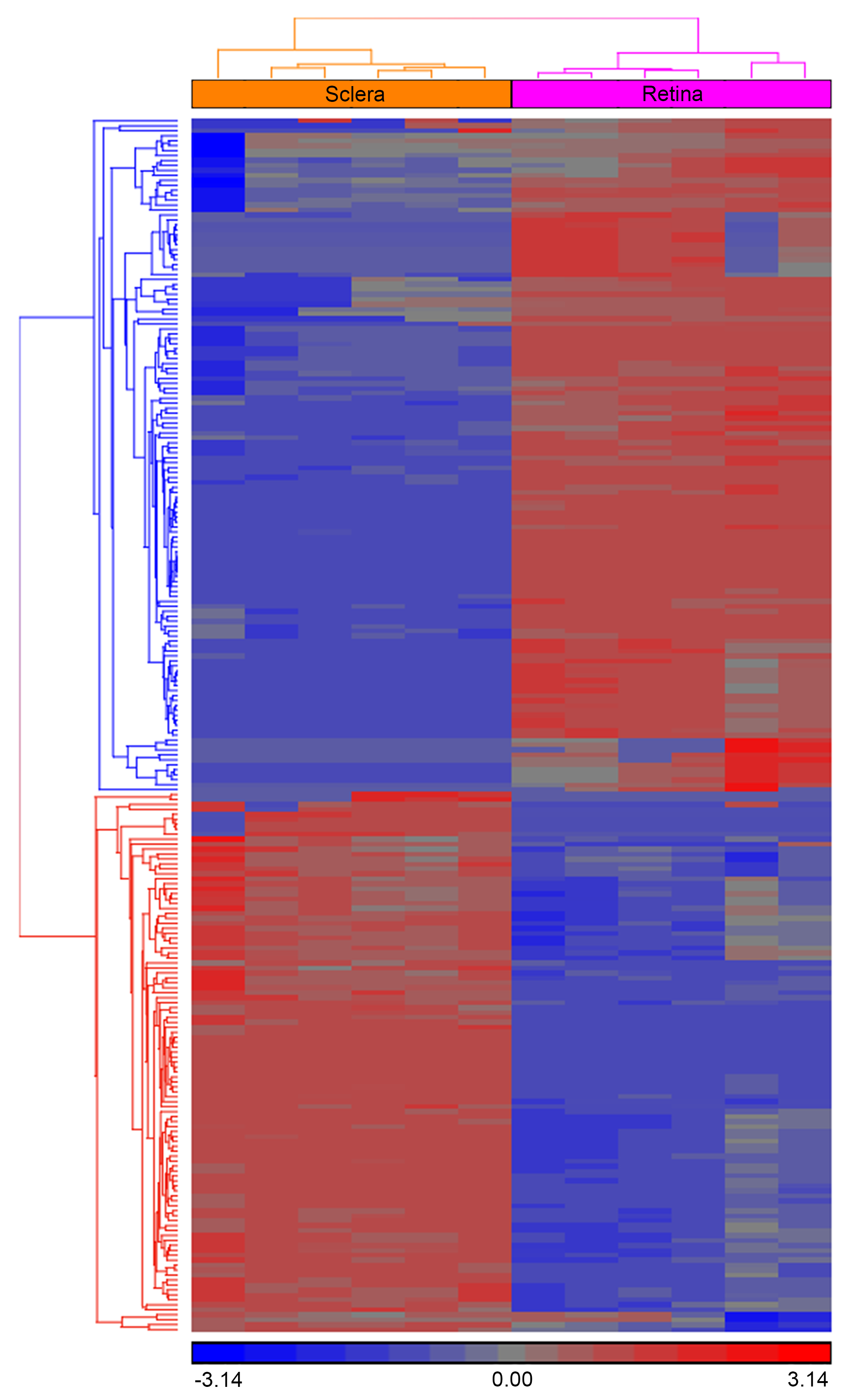

Supplement: S1 Fig — Logarithmic values (base 2) of Agilent total gene signal for differentially expressed miRNAs (cutoff: FC > 2, FDR-adjusted p-value < 0.05) were quantile normalized, shifted to mean zero, scaled to standard deviation of 1.0 and subjected to hierarchical clustering using Euclidean dissimilarity and average linkage. The color scale indicates transcript abundance: red identifies an increase in relative miRNA abundance; blue identifies a decrease in relative miRNA abundance. Columns show individual samples, whereas rows show individual miRNAs. (TIF) [file pone.0162541.s001.tif]

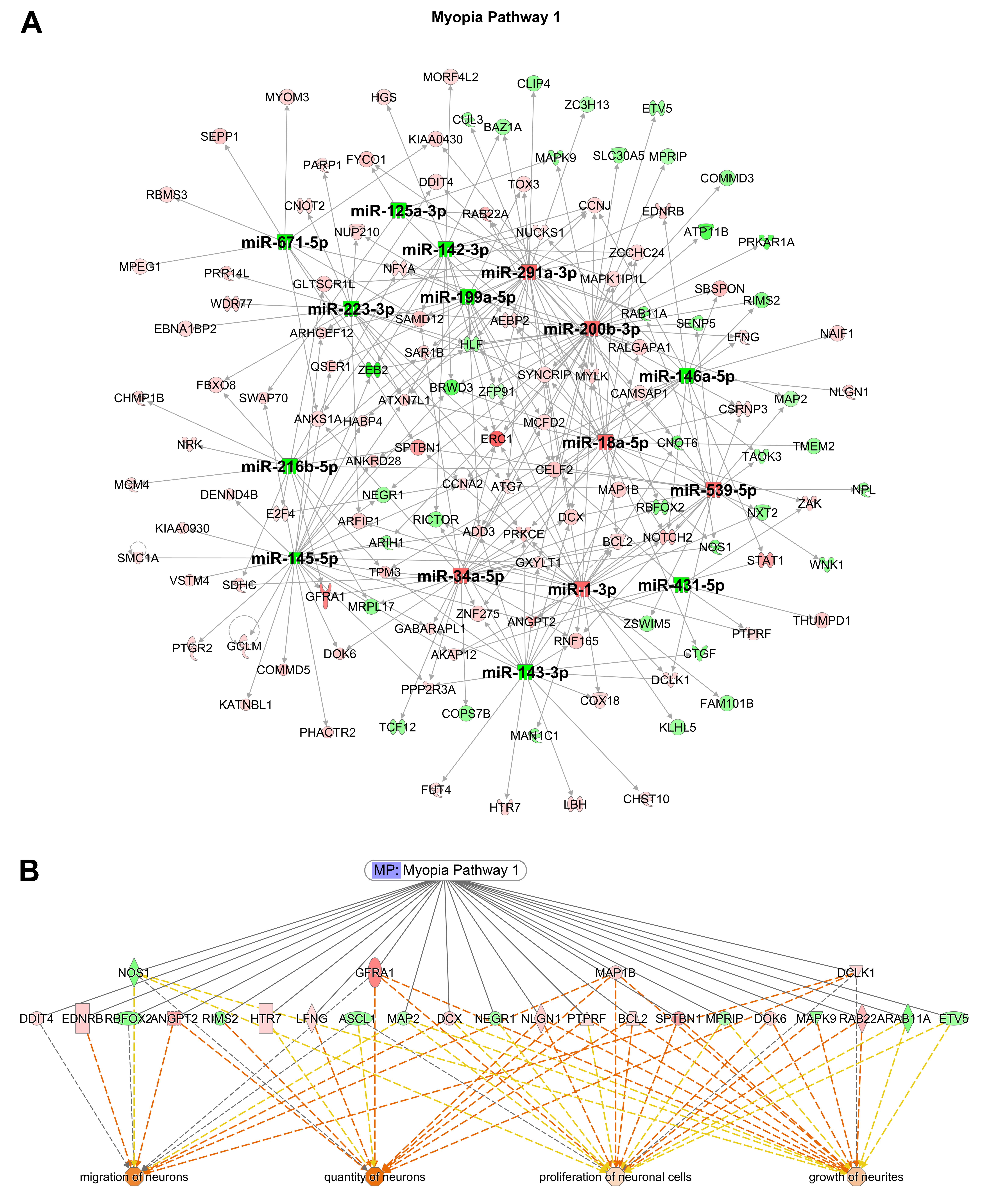

Supplement: S2 Fig — (A) Diagram showing interactions between miRNAs differentially expressed in myopic retina and their target mRNAs. Arrows show relationships between different miRNAs and mRNAs. (B) Overlap between signaling pathway #1 and top gene ontology categories. Red identifies genes/categories which are up-regulated in myopic retina, whereas green identifies genes/categories which are down-regulated in myopic retina. (TIF) [file pone.0162541.s002.tif]

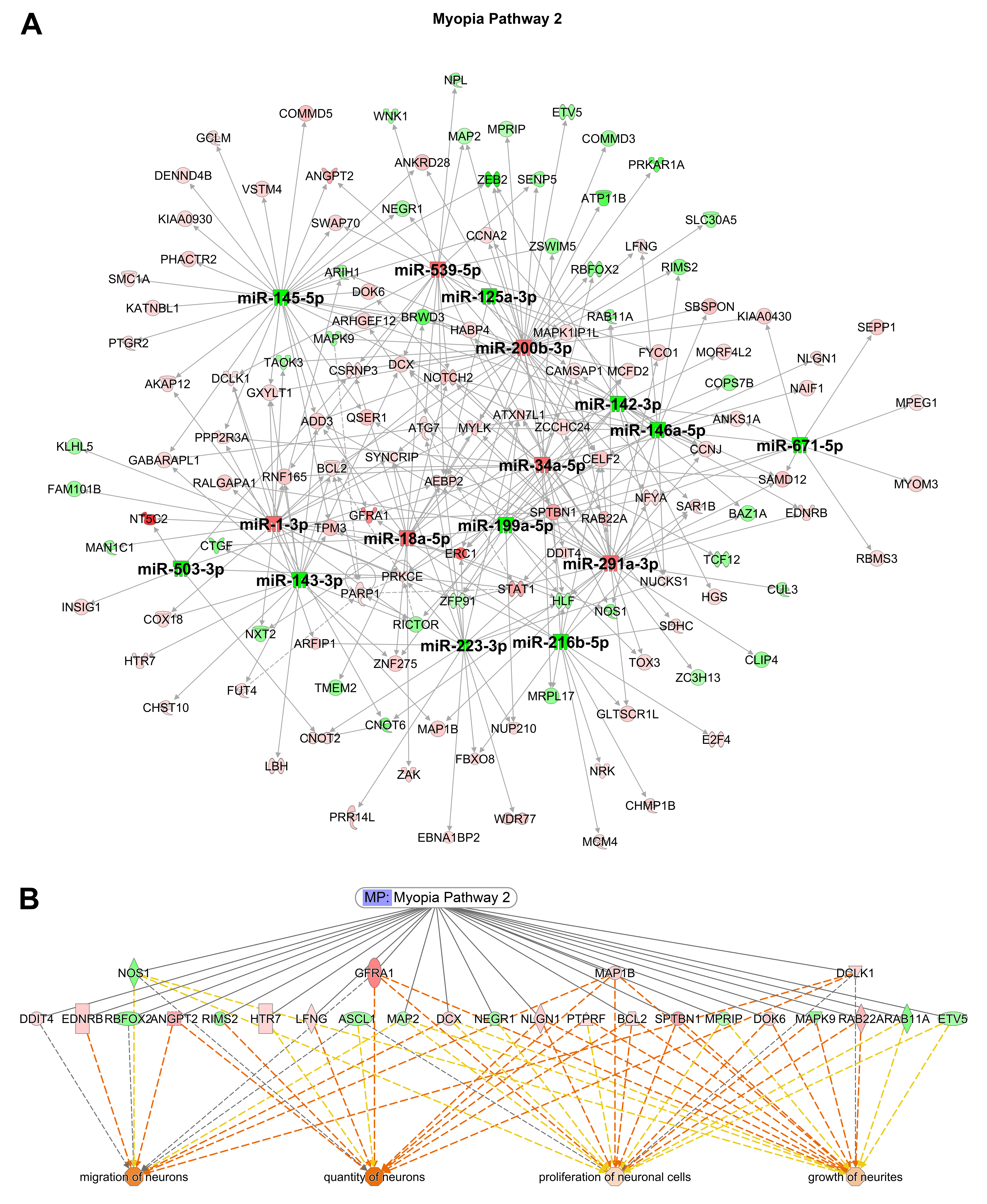

Supplement: S3 Fig — (A) Diagram showing interactions between miRNAs differentially expressed in myopic retina and their target mRNAs. Arrows show relationships between different miRNAs and mRNAs. (B) Overlap between signaling pathway #2 and top gene ontology categories. Red identifies genes/categories which are up-regulated in myopic retina, whereas green identifies genes/categories which are down-regulated in myopic retina. (TIF) [file pone.0162541.s003.tif]

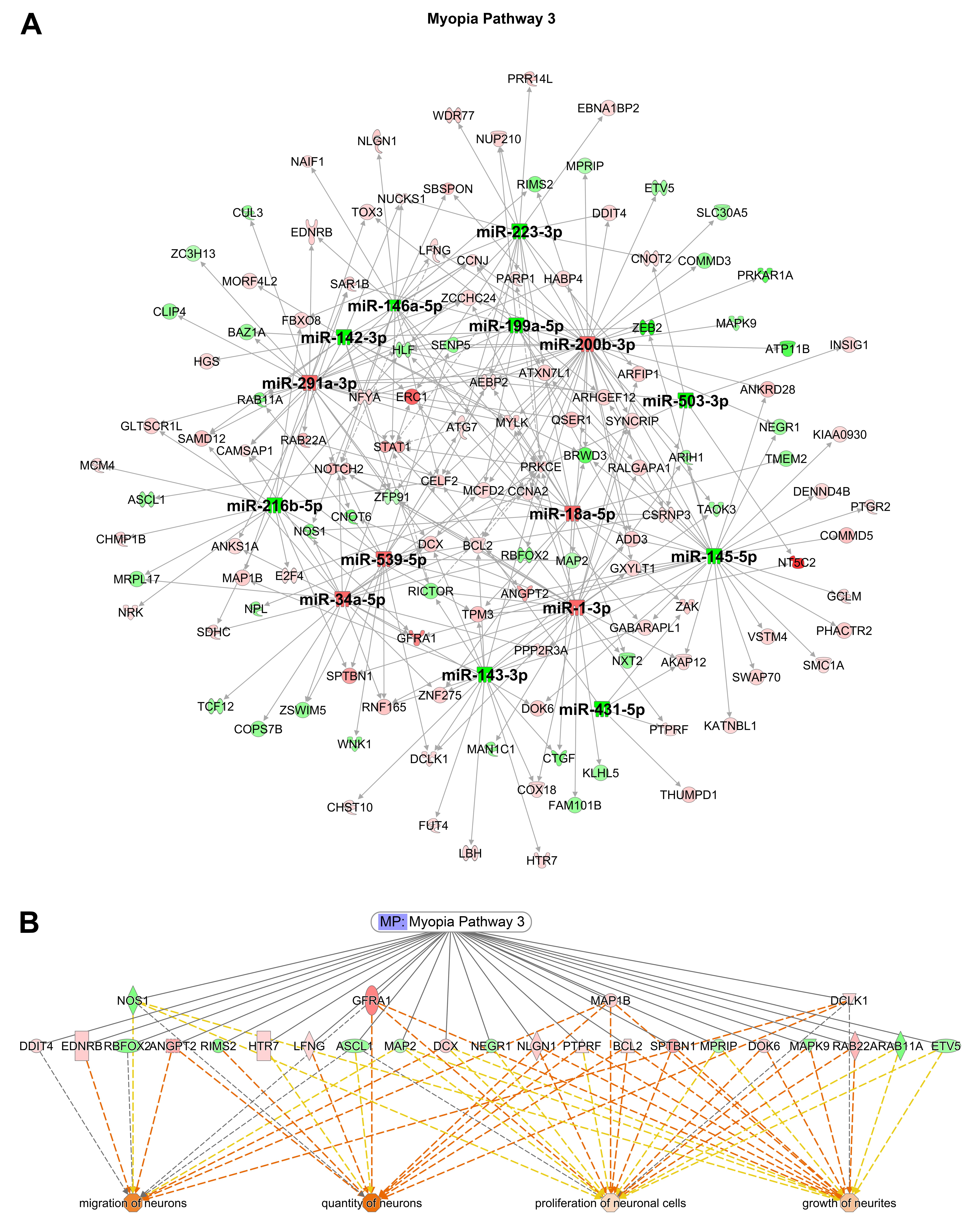

Supplement: S4 Fig — (A) Diagram showing interactions between miRNAs differentially expressed in myopic retina and their target mRNAs. Arrows show relationships between different miRNAs and mRNAs. (B) Overlap between signaling pathway #3 and top gene ontology categories. Red identifies genes/categories which are up-regulated in myopic retina, whereas green identifies genes/categories which are down-regulated in myopic retina. (TIF) [file pone.0162541.s004.tif]

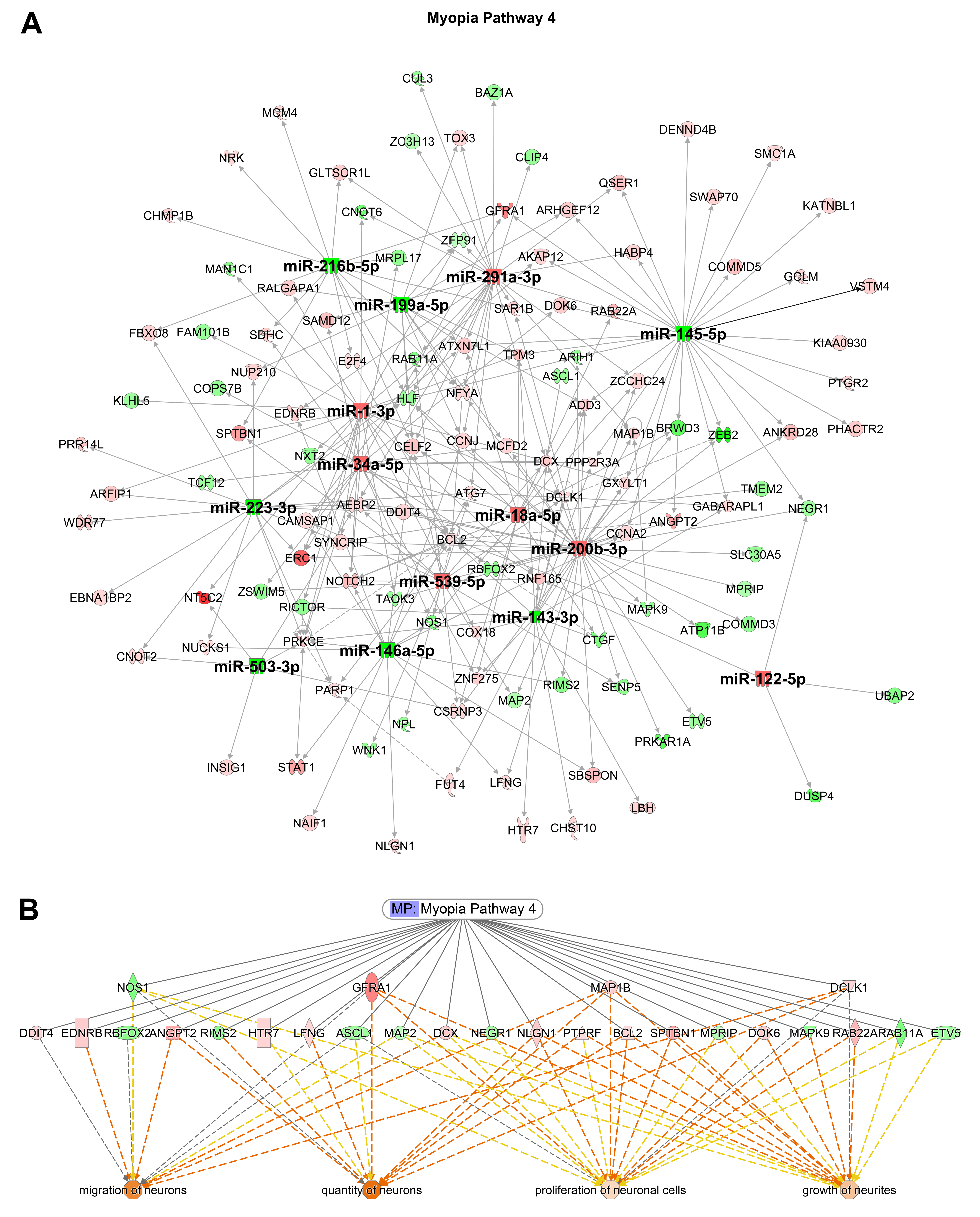

Supplement: S5 Fig — (A) Diagram showing interactions between miRNAs differentially expressed in myopic retina and their target mRNAs. Arrows show relationships between different miRNAs and mRNAs. (B) Overlap between signaling pathway #4 and top gene ontology categories. Red identifies genes/categories which are up-regulated in myopic retina, whereas green identifies genes/categories which are down-regulated in myopic retina. (TIF) [file pone.0162541.s005.tif]

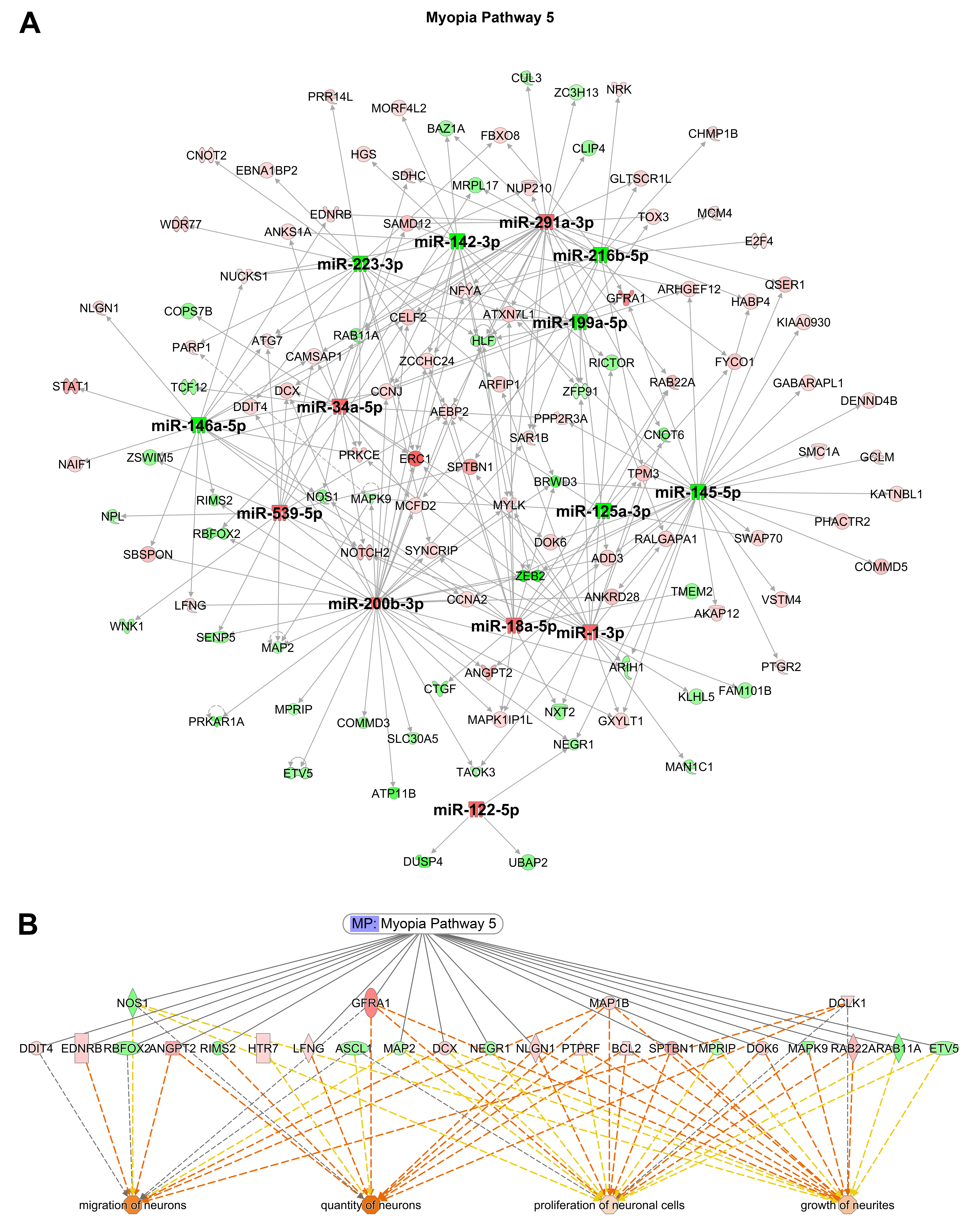

Supplement: S6 Fig — (A) Diagram showing interactions between miRNAs differentially expressed in myopic retina and their target mRNAs. Arrows show relationships between different miRNAs and mRNAs. (B) Overlap between signaling pathway #5 and top gene ontology categories. Red identifies genes/categories which are up-regulated in myopic retina, whereas green identifies genes/categories which are down-regulated in myopic retina. (TIF) [file pone.0162541.s006.tif]

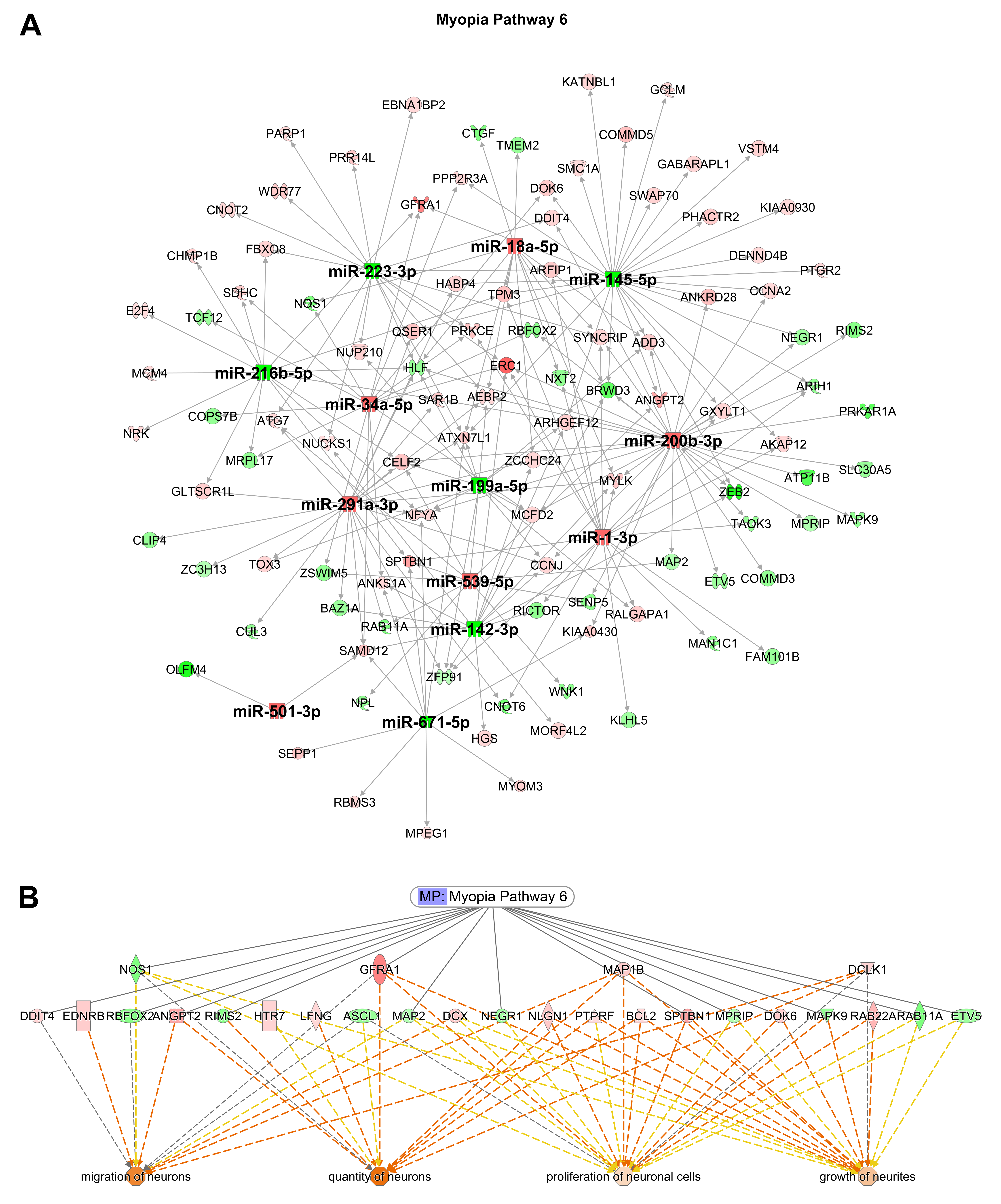

Supplement: S7 Fig — (A) Diagram showing interactions between miRNAs differentially expressed in myopic retina and their target mRNAs. Arrows show relationships between different miRNAs and mRNAs. (B) Overlap between signaling pathway #6 and top gene ontology categories. Red identifies genes/categories which are up-regulated in myopic retina, whereas green identifies genes/categories which are down-regulated in myopic retina. (TIF) [file pone.0162541.s007.tif]

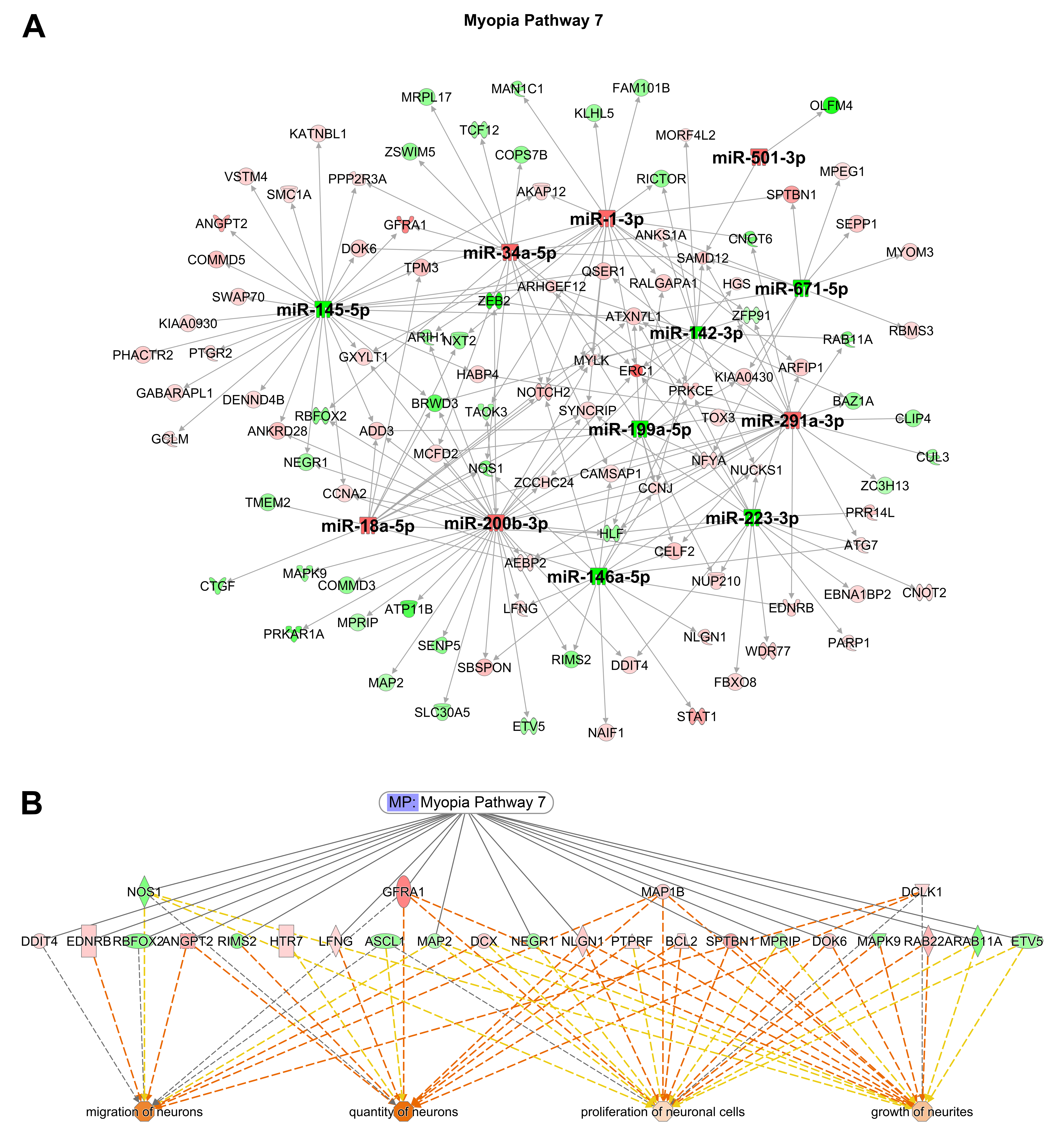

Supplement: S8 Fig — (A) Diagram showing interactions between miRNAs differentially expressed in myopic retina and their target mRNAs. Arrows show relationships between different miRNAs and mRNAs. (B) Overlap between signaling pathway #7 and top gene ontology categories. Red identifies genes/categories which are up-regulated in myopic retina, whereas green identifies genes/categories which are down-regulated in myopic retina. (TIF) [file pone.0162541.s008.tif]

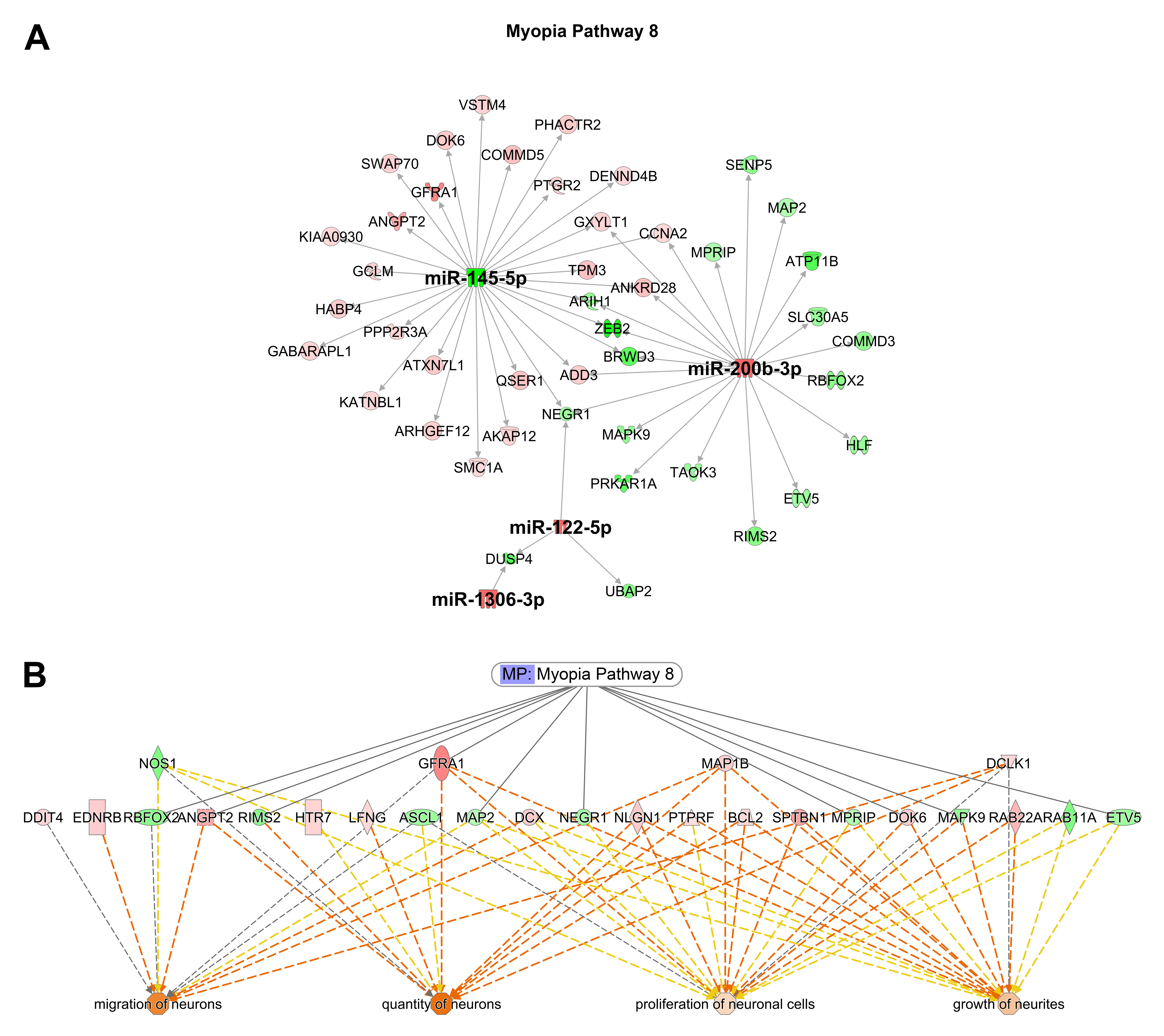

Supplement: S9 Fig — (A) Diagram showing interactions between miRNAs differentially expressed in myopic retina and their target mRNAs. Arrows show relationships between different miRNAs and mRNAs. (B) Overlap between signaling pathway #8 and top gene ontology categories. Red identifies genes/categories which are up-regulated in myopic retina, whereas green identifies genes/categories which are down-regulated in myopic retina. (TIF) [file pone.0162541.s009.tif]

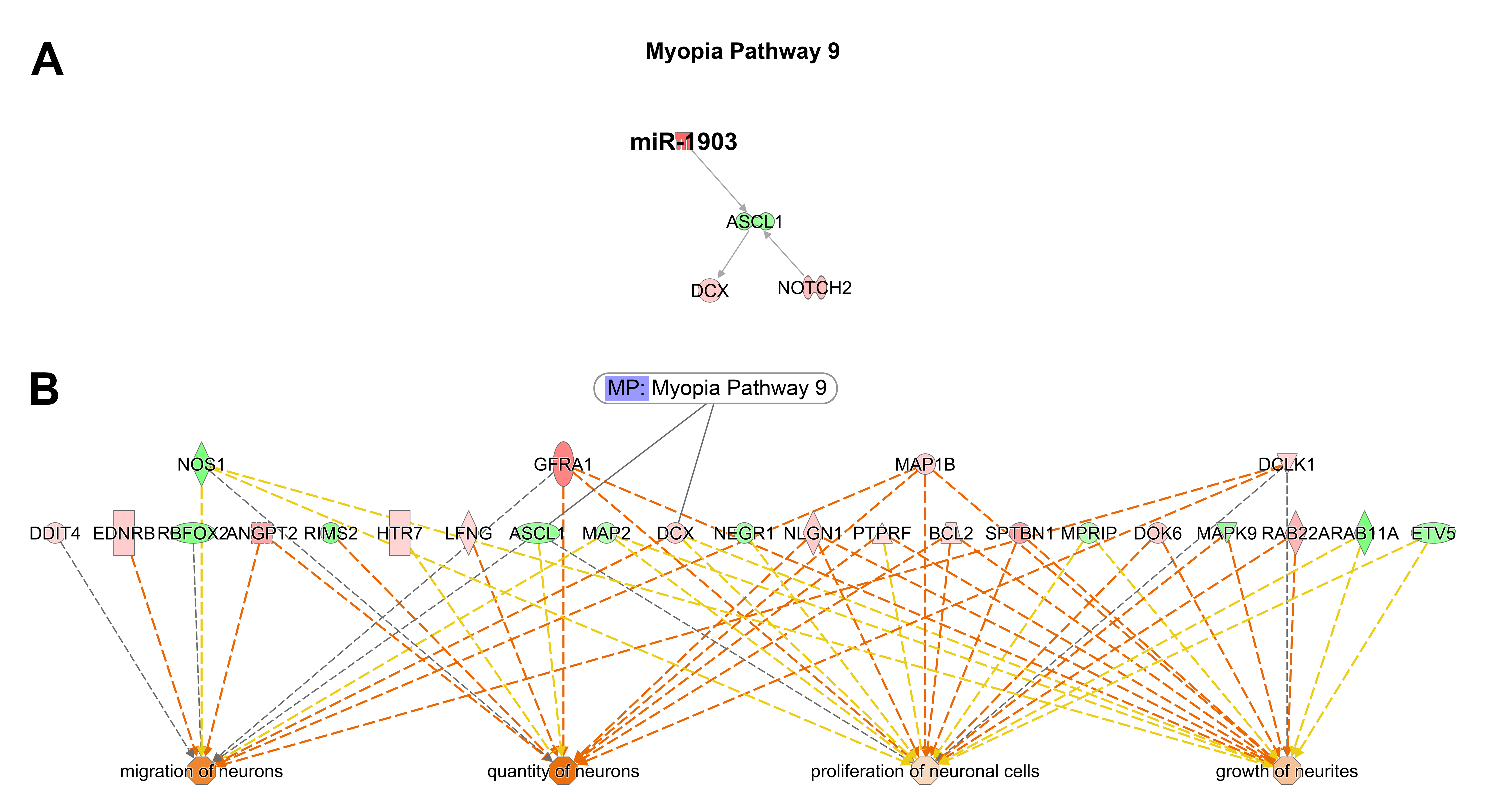

Supplement: S10 Fig — (A) Diagram showing interactions between miRNAs differentially expressed in myopic retina and their target mRNAs. Arrows show relationships between different miRNAs and mRNAs. (B) Overlap between signaling pathway #9 and top gene ontology categories. Red identifies genes/categories which are up-regulated in myopic retina, whereas green identifies genes/categories which are down-regulated in myopic retina. (TIF) [file pone.0162541.s010.tif]
