# Supplementary material for: Large-Scale microRNA Expression Profiling Identifies Putative Retinal miRNA-mRNA Signaling Pathways Underlying Form-Deprivation Myopia in Mice
Source: PLoS One. 2016 Sep 13;11(9):e0162541. doi: 10.1371/journal.pone.0162541 (PMC5021328; doi:10.1371/journal.pone.0162541)
Supplement: S3 Table — (DOCX) [file pone.0162541.s013.docx]

**Table S3 MiRNAs without target mRNAs differentially expressed in myopic retina versus control retina**

| **miRBase ID** | **miRNA name** | **miRNA cluster** | **Fold change** | ***P*-value** |
| --- | --- | --- | --- | --- |
| MIMAT0009413 | mmu-miR-1947-5p | miR-1947-5p (miRNAs w/seed GGACGAG) | 31.5 | 1.47 × 10^-04^ |
| MIMAT0004619 | mmu-miR-200a-5p | miR-200a-5p (and other miRNAs w/seed AUCUUAC) | 18.8 | 9.46 × 10^-05^ |
| MIMAT0004533 | mmu-miR-141-5p | miR-141-5p (and other miRNAs w/seed AUCUUCC) | 13.9 | 4.75 × 10^-06^ |
| MIMAT0004871 | mmu-miR-465b-5p | miR-465b-5p (and other miRNAs w/seed AUUUAGA) | 12.8 | 5.93 × 10^-04^ |
| MIMAT0004664 | mmu-miR-214-5p | miR-214-5p (miRNAs w/seed GCCUGUC) | 12.6 | 8.27 × 10^-03^ |
| MIMAT0009400 | mmu-miR-1936 | miR-1936 (miRNAs w/seed AACUGAC) | 12.3 | 9.56 × 10^-06^ |
| MIMAT0004881 | mmu-miR-466f-5p | miR-466f-5p (miRNAs w/seed ACGUGUG) | 11.5 | 3.85 × 10^-03^ |
| MIMAT0009421 | mmu-miR-669o-5p | miR-669o-5p (miRNAs w/seed AGUUGUG) | 10.9 | 2.18 × 10^-03^ |
| MIMAT0003483 | mmu-miR-696 | miR-696 (miRNAs w/seed CGUGUGC) | 9.0 | 1.28 × 10^-03^ |
| MIMAT0004628 | mmu-miR-21-3p | miR-21-3p (and other miRNAs w/seed AACAGCA) | 8.9 | 8.01 × 10^-05^ |
| MIMAT0004824 | mmu-miR-673-3p | miR-673-3p (and other miRNAs w/seed CCGGGGC) | 8.4 | 1.65 × 10^-05^ |
| MIMAT0011212 | mmu-miR-2136 | miR-2136 (miRNAs w/seed UGGGUGU) | 6.3 | 5.02 × 10^-04^ |
| MIMAT0004841 | mmu-miR-871-5p | miR-743a-5p (and other miRNAs w/seed AUUCAGA) | 5.8 | 5.51 × 10^-04^ |
| MIMAT0004526 | mmu-miR-101a-5p | miR-101a-5p (miRNAs w/seed CAGUUAU) | 5.1 | 1.88 × 10^-04^ |
| MIMAT0007873 | mmu-miR-1896 | miR-1896 (miRNAs w/seed UCUCUGA) | 4.9 | 1.66 × 10^-03^ |
| MIMAT0004885 | mmu-miR-467c-5p | miR-467c-5p (and other miRNAs w/seed AAGUGCG) | 4.6 | 1.68 × 10^-04^ |
| MIMAT0004884 | mmu-miR-466h-5p | miR-669m-5p (and other miRNAs w/seed GUGUGCA) | 4.3 | 1.29 × 10^-03^ |
| MIMAT0003476 | mmu-miR-669b-5p | miR-669b-5p (miRNAs w/seed GUUUUGU) | 4.3 | 1.02 × 10^-03^ |
| MIMAT0005853 | mmu-miR-669e-5p | miR-331-5p (and other miRNAs w/seed GUCUUGU) | 4.2 | 2.41 × 10^-03^ |
| MIMAT0004856 | mmu-miR-105 | miR-105 (miRNAs w/seed CAAGUGC) | 4.2 | 1.83 × 10^-04^ |
| MIMAT0003494 | mmu-miR-704 | miR-704 (miRNAs w/seed GACAUGU) | 4.0 | 3.00 × 10^-04^ |
| MIMAT0000372 | mmu-miR-294-3p | miR-291a-3p (and other miRNAs w/seed AAGUGCU) | 3.9 | 3.79 × 10^-03^ |
| MIMAT0004626 | mmu-miR-18a-3p | miR-18a-3p (and other miRNAs w/seed CUGCCCU) | 3.9 | 6.01 × 10^-04^ |
| MIMAT0009427 | mmu-miR-669n | miR-5010-3p (and other miRNAs w/seed UUUGUGU) | 3.3 | 7.22 × 10^-03^ |
| MIMAT0004647 | mmu-miR-338-5p | miR-338-5p (miRNAs w/seed ACAAUAU) | 3.2 | 4.09 × 10^-04^ |
| MIMAT0000380 | mmu-miR-302a-3p | miR-291a-3p (and other miRNAs w/seed AAGUGCU) | 2.4 | 5.27 × 10^-04^ |
| MIMAT0014816 | mmu-miR-3099-3p | miR-3099 (and other miRNAs w/seed AGGCUAG) | 2.4 | 7.47 × 10^-03^ |
| MIMAT0000137 | mmu-miR-126-5p | miR-126a-5p (and other miRNAs w/seed AUUAUUA) | -2.3 | 3.67 × 10^-03^ |
| MIMAT0004572 | mmu-miR-290-3p | miR-467a-5p (and other miRNAs w/seed AAGUGCC) | -2.4 | 6.43 × 10^-03^ |
| MIMAT0003782 | mmu-miR-676-3p | miR-676 (and other miRNAs w/seed CGUCCUG) | -2.7 | 9.35 × 10^-04^ |
| MIMAT0004640 | mmu-miR-325-3p | miR-325-3p (miRNAs w/seed UUAUUGA) | -2.8 | 5.59 × 10^-04^ |
| MIMAT0003742 | mmu-miR-455-3p | miR-455-3p (miRNAs w/seed CAGUCCA) | -3.1 | 1.74 × 10^-03^ |
